# Unveiling sex dimorphism in the healthy cardiac anatomy: Fundamental differences between male and female heart shapes

**DOI:** 10.1113/JP288667

**Published:** 2025-09-25

**Authors:** Beatrice Moscoloni, Cameron Beeche, Julio A. Chirinos, Patrick Segers, Mathias Peirlinck

**Affiliations:** ^1^ Department of Biomechanical Engineering Delft University of Technology Delft The Netherlands; ^2^ BioMMeda – Institute for Biomedical Engineering and Technology Ghent University Ghent Belgium; ^3^ Division of Cardiovascular Medicine Hospital of the University of Pennsylvania Philadelphia PA USA; ^4^ Department of Bioengineering University of Pennsylvania Philadelphia PA USA

**Keywords:** anthropometrics, cardiac anatomy, confounding factors, sex differences, statistical shape modelling

## Abstract

**Abstract:**

Sex‐based differences in cardiovascular disease are well documented, yet the precise nature and extent of these discrepancies in cardiac anatomy remain incompletely understood. Traditional scaling models often fail to capture the interplay of age, blood pressure and body size, prompting a more nuanced investigation. Here we use statistical shape modelling in a healthy subset (*n* = 456) of the UK Biobank to explore sex‐specific variations in biventricular anatomy. We reconstruct 3D meshes and perform multivariate analyses of shape coefficients, controlling for age, blood pressure and various body size metrics. Our findings reveal that sex alone explains at least 25% of morphological variability, with strong discrimination between men and women (AUC = 0.96–0.71) persisting even after correction for confounders. Notably, the most discriminative modes highlight pronounced differences in cardiac chamber volumes, the anterior–posterior width of the right ventricle and the relative positioning of the cardiac chambers. These results underscore that sex has a fundamental influence on cardiac morphology, which may have important clinical implications for differing cardiac structural assessments in men and women. Future work should investigate how these anatomical differences manifest in various cardiovascular conditions, ultimately paving the way for more precise risk stratification and personalised therapeutic strategies for both men and women.

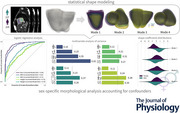

**Key points:**

- Men's and women's hearts differ significantly in overall shape and size, but an in‐depth quantification of these sex differences in healthy cardiac anatomy is lacking.
- We used a three‐dimensional statistical shape modelling approach that goes beyond standard clinical measurements to capture subtle anatomical features.
- Our findings show that sex alone accounts for at least 25% of the natural variation in heart structure, even after correcting for age, blood pressure and various body size metric confounders.
- Female hearts consistently present smaller chambers and different inter‐chamber positioning compared with male hearts.
- Our findings highlight the importance of sex‐specific anatomical insights for better diagnosis, treatment and research on heart disease.

## Graphical abstract

**Abstract figure legend** We present a shape modelling‐based morphological analysis of sex differences in cardiac anatomy. We conduct our analysis on 456 healthy subjects from the UK Biobank (227M/229F) to uncover sex‐based differences in healthy cardiac morphology. Our analysis shows that sex differences in cardiac anatomy are not limited to average shape but span the entire morphological distribution. In addition, sex consistently accounts for at least 25% of the variability, even after accounting for age, systolic blood pressure and body size. Finally, no confounder correction scheme fully eliminates geometric differences between male and female hearts. Moving forward, accounting for these intrinsic differences in clinical biomarkers and imaging‐based diagnostic thresholds will be essential to reduce sex‐related disparities in cardiovascular care.

## Introduction

Cardiovascular disease (CVD) remains the leading cause of death among women, accounting for one in three female deaths worldwide (Chang et al., 2023; Mehta et al., 2022). Despite increasing awareness of sex differences in CVD presentation, pathophysiology and response to treatment, minimal progress has been achieved in reducing heart disease among women (Vaccarezza et al., 2020). One contributing factor to these disparities is the historical underrepresentation of women in clinical trials (Daitch et al., 2022; Sosinsky et al., 2022), which has led to persistent knowledge gaps regarding sex dimorphism in cardiac physiology and pathophysiology (St. Pierre et al., 2022). This lack of insight on sex differences in cardiac anatomy, function and microstructure hinders the development of sex‐specific diagnostic criteria and treatment guidelines (Martin & Leinwand, 2024; Peirlinck et al., 2021). Although awareness of sex differences in cardiac anatomy is slowly growing, women are predominantly diagnosed based on sex‐agnostic criteria that may bias care towards male‐centric standards (St. Pierre et al., 2024; Vaccarezza et al., 2020). As a result, women with heart disease are often under‐treated, under‐diagnosed and experience worse outcomes compared to men (Beale et al., 2018). Furthermore, variability in healthy cardiac anatomy has been hypothesised to underpin sex‐specific patterns of pathological remodelling (Beale et al., 2018; Peirlinck et al., 2019), yet the extent and nature of this structural variability remain poorly understood (Martin & Leinwand, 2024). For instance while female hearts are generally smaller, recent findings suggest that differences in cardiac geometry are not solely attributable to scale but reflect unique patterns that do not linearly correspond to those observed in males. In addition, these disparities are further compounded by other confounding factors such as age, body size and blood pressure (Hendriks et al.,; St. Pierre et al., 2022; Westaby et al., 2023). Recent literature studies sought to quantify these sex differences and examine their correlation with confounding variables, such as body size and age (Gao et al., 2019; Ji et al., 2022; Westaby et al., 2023). However many of these investigations rely on retrospective analyses of heterogeneous cohorts, making it challenging to draw meaningful conclusions (St. Pierre et al., 2022). Moreover, shape and anatomical assessments in these studies, and more generally in clinical practice, often rely on simple two‐dimensional morphological parameters, such as lengths and diameters (Westaby et al., 2023). Such approaches overlook the rich data provided by current imaging techniques and underestimate the intrinsic complexity of global and local shape features in cardiac anatomy, and its impact on clinical biomarkers (Bruse et al., 2016; Mincholé et al., 2019; Shiwani et al., 2025). For instance a recent population‐based study challenged the *one‐size‐fits‐all* threshold of ≥15 mm for maximum wall thickness in hypertrophic cardiomyopathy diagnosis, showing a substantial sex skew in clinical classification (Shiwani et al., 2025). Similarly ventricular geometry has been shown to significantly affect electrocardiography (ECG) biomarkers, underscoring the need to account for anatomical variation in ECG‐based diagnostic models (Mincholé et al., 2019). Consequently a comprehensive morphological analysis that accounts for potential confounding variables is crucial for advancing our understanding of sex differences in cardiac morphology, and their implications for diagnosis and treatment of CVD (Gao et al., 2019). In this context, combining computer vision approaches and data‐driven morphological analysis methods with large population biobanks provides an ideal framework for conducting robust, large‐scale morphological studies that capture the complexity of sex differences in cardiac anatomy. Statistical shape models, often paired with deep learning‐based segmentation, have been used to simultaneously analyse complex three‐dimensional global and regional shape patterns (Burns et al., 2024; Govil, Crabb et al., 2023; Govil, Mauger et al., 2023; Mauger et al., 2019). By leveraging registration, mapping and dimensionality reduction techniques on large cohorts of anatomical shapes, these models build an anatomical atlas that effectively encodes cohort‐wide anatomical variability through a relatively small set of principal components (Cutugno et al., 2021; Mauger et al., 2019; Rodero et al., 2021; Verstraeten et al., 2024). In turn, this atlas enables a more complex and comprehensive data‐driven analysis of morphological variation than is possible with traditional measurements (Govil, Mauger et al., 2023; Suinesiaputra et al., 2018). For example a recent large‐scale study constructed a heart shape atlas from UK Biobank participants to identify genetic determinants of cardiac shape and associate specific shape components to increased cardiometabolic disease risk (Burns et al., 2024). Nevertheless a dedicated statistical shape analysis explicitly addressing sex differences in cardiac morphology within a healthy population is still lacking.

In this work we apply a data‐driven morphological analysis to investigate sex differences in cardiac anatomy within a healthy subset of the UK Biobank imaging population (Littlejohns et al., 2020). Our approach employs deep learning to automatically segment the cardiac structures from cardiac magnetic resonance (CMR) images, followed by statistical shape modelling to obtain detailed three‐dimensional morphological features. By coupling this approach with data‐driven regression techniques, we:

- Examine how morphological descriptors differ between the male and female subgroups.
- Quantify the impact of sex on cardiac morphology, after accounting for age, body size and blood pressure.
- Identify the cardiac shape components that best distinguish male and female populations, after accounting for confounders.

## Methods

**Figure 1:**
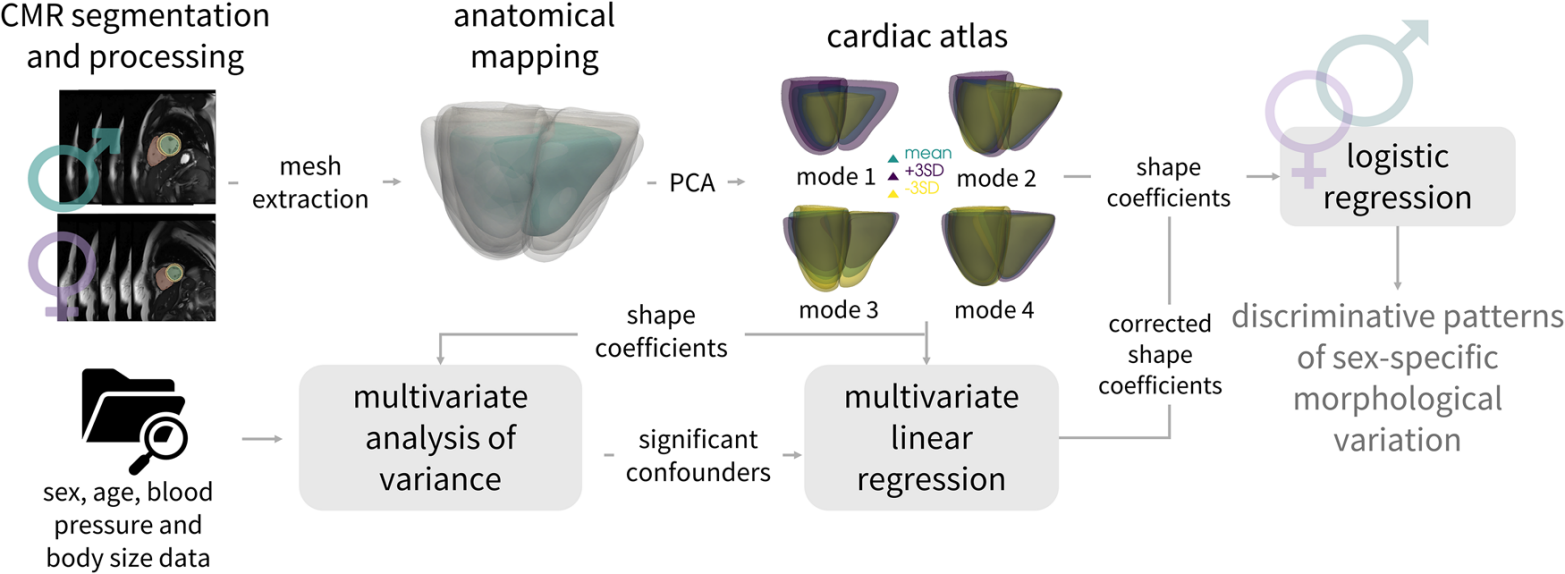
Statistical shape analysis pipeline for assessing sex‐specific cardiac anatomical variability Our pipeline includes magnetic resonance imaging processing and segmentation, anatomical mapping, multivariate analysis of covariance and regression techniques to identify discriminative patterns of sex‐specific morphological variation, accounting for anthropometrics, demographics and physiological confounding factors.

We introduce a statistical shape analysis pipeline to investigate sex‐specific cardiac anatomical variability and its interaction with demographic and physiological confounding factors of cardiac morphology (Fig. 1). Our pipeline integrates medical image processing, deep learning‐based segmentation, statistical shape modelling, multivariate analysis and regression techniques to comprehensively characterise sex differences in cardiac morphology. We applied our pipeline on CMR images from a subset of healthy participants from the UK Biobank to assess the effect of sex, age, body size and blood pressure on cardiac anatomy. Finally we identified the most discriminating patterns of sex‐specific morphological variation after correction for other significant confounding variables.

### Cohort and imaging dataset

#### Population selection and CMR imaging dataset

We leveraged the UK Biobank imaging population, which includes ∼ 100,000 participants who underwent CMR imaging (Littlejohns et al., 2020). From these we selected 456 healthy subjects with no reported history of CVD, hypertension, respiratory disease, diabetes mellitus, hyperlipidemia, hematologic disease, renal disease, rheumatologic disease, malignancy, symptoms of chest pain or dyspnoea (Petersen et al., 2016). To rule out additional risk factors, we further excluded current or former smokers and individuals with body mass index (BMI) ≥30 kg/m. Although these criteria generally ensure normotensive subjects, we further screened subjects' diastolic and systolic blood pressures, excluding those with a single reading greater than 180/110 mmHg in line with the 2020 global hypertension practice guidelines of the International Society of Hypertension for diagnosis at a single visit (Unger et al., 2020). The resulting final healthy subset includes 227 males and 229 females. A summary of their main anthropometric, demographic and functional variables is provided in Table 1.

**Table 1:** Anthropometric, demographic and functional variables of the dataset. Continuous variables are expressed as mean ± standard deviation.

Type	Subject data	Male (*n*=227)	Female (*n*=229)	*P*‐value
**Demographics**	Age (years)	60.1 ± 7.6	60.1 ± 7.6	0.9544
**Anthropometrics**	Body mass index (kg/m 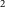 )	24.94 ± 2.55	23.89 ± 2.77	<0.0001
	Body surface area (m 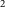 )	1.94 ± 0.15	1.70 ± 0.13	<0.0001
	Standing height (cm)	176.10 ± 6.85	164.00 ± 6.45	<0.0001
	Weight (kg)	77.45 ± 9.99	64.28 ± 7.45	<0.0001
**Blood pressure**	Systolic blood pressure (mmHg)	133.25 ± 15.11	125.45 ± 17.18	<0.0001
	Diastolic blood pressure (mmHg)	77.61 ± 10.01	72.47 ± 9.44	<0.0001
	Mean arterial pressure (mmHg)	96.16 ± 10.49	90.13 ± 11.07	<0.0001
**Cardiac function**	End diastolic volume (ml)	155.52 ± 30.43	121.34 ± 24.11	<0.0001
	End systolic volume (ml)	69.81 ± 16.87	51.95 ± 12.64	<0.0001
	Stroke volume (ml)	85.70 ± 18.82	69.35 ± 14.89	<0.0001
	Cardiac index (L/min/m^2^)	2.63 ± 0.51	2.51 ± 0.47	0.0275
	Cardiac output (L/min)	5.09 ± 1.08	4.28 ± 0.92	<0.0001
	Ejection fraction (%)	55.12 ± 5.97	57.25 ± 5.25	0.0003
	Heart rate (bpm)	60.29 ± 8.99	62.23 ± 8.68	0.0243

The complete CMR protocol of the UK Biobank has already been described in detail elsewhere (Petersen et al., 2016). Briefly it consists of three steady‐state free precession cine images in long axis views, and a complete short axis steady‐state free precession cine stack covering both the right ventricle (RV) and the left ventricle (LV). Each slice was acquired in a separate breath‐hold. We use the end‐diastolic phase CMR image data from the 456 selected subjects, including the stack of short‐axis 2D views, and the single long‐axis horizontal view, also referred to as the four‐chamber view. Both the short‐ and long‐axis images have an in‐plane resolution of 1.8×1.8, mm^2^. Furthermore the short‐axis image stack typically comprises 10 slices with a slice thickness of 8.0 mm (Littlejohns et al., 2020).

#### CMR segmentation and preprocessing

**Figure 2:**
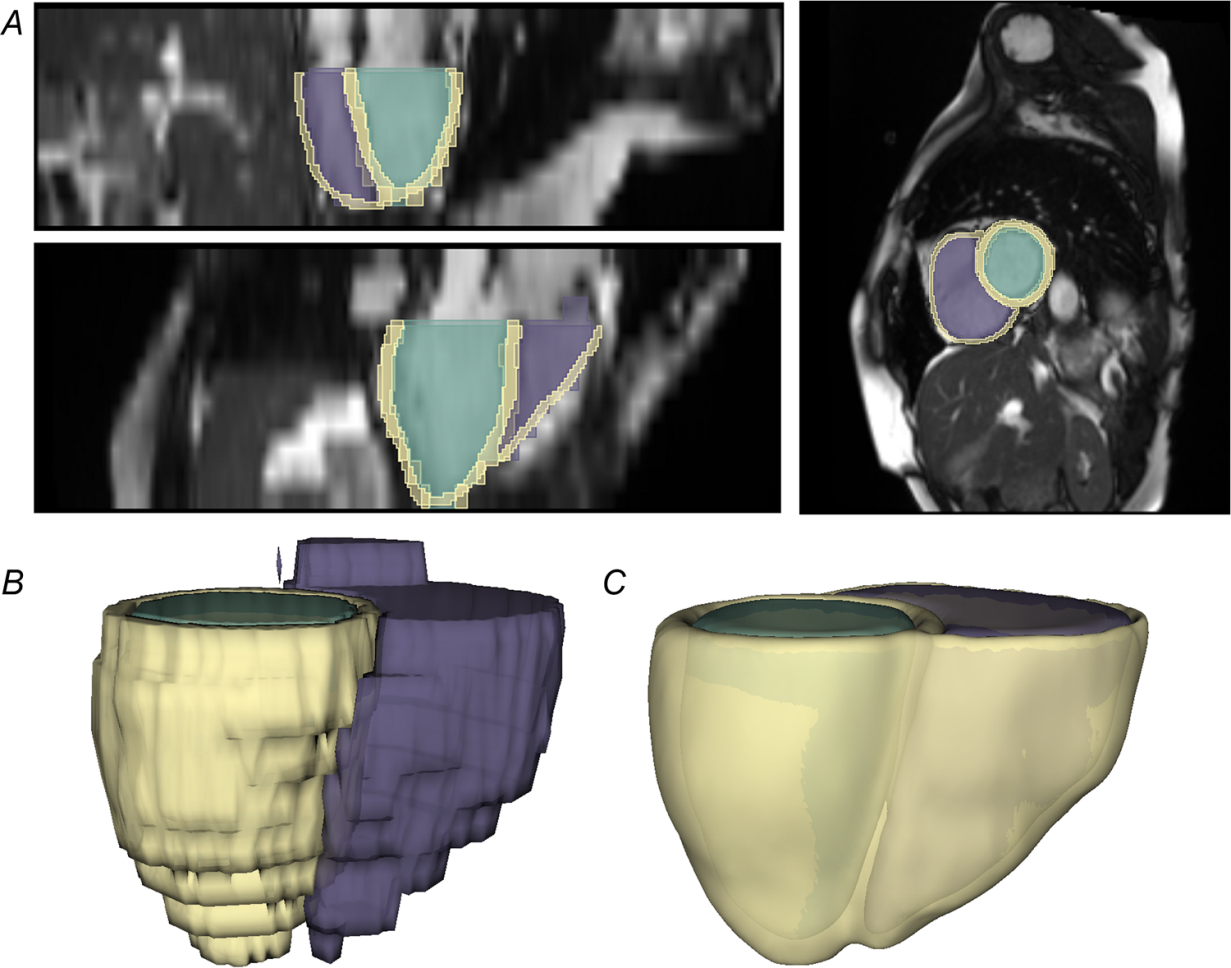
Pipeline for CMR segmentation and preprocessing *A*, Overlay of the original low‐resolution and high‐resolution segmentations on representative long‐axis (left) and short‐axis (right) cardiac magnetic resonance images. *B*, Volume reconstruction of our low‐resolution segmentation obtained from the fully convolutional neural network. *C*, Volume reconstruction of the final high‐resolution segmentation obtained through the β‐variational autoencoder generative model and processing steps. Reproduced by kind permission of UK Biobank ©.

Figure 2 provides an overview of our CMR segmentation and preprocessing pipeline. This process was aimed at obtaining consistent, high‐resolution segmentation of the cardiac structures of interest, required for the downstream shape analysis; it can be summarised in three steps.

**Step 1 – Initial segmentation**: We applied a pre‐trained, openly available fully convolutional neural network (Bai et al., 2018) to segment five cardiac structures of interest: the left ventricular (LV) and right ventricular (RV) blood pools, the LV myocardium, and left and right atrial blood pools. Specifically we segmented the LV/RV blood pool and LV myocardium on both short‐axis and long‐axis slices, while atrial blood pools were segmented solely on the long‐axis slices. Fully convolutional neural networks (Bai et al., 2018) achieve pixel‐wise image segmentation by using multiple convolutional filters and softmax regression to assign probabilistic label maps to each pixel of the image. The final segmentation is determined at each pixel by the label class with the highest softmax probability. Although the resulting short‐axis segmentations allow for volume reconstructions, they still suffer from anisotropic low‐resolution, motion artefacts and potential topological abnormalities as shown in Fig. 2*B* (Pentenga et al., 2023; Wang et al., 2021). Hence we performed further processing on the segmented data.

**Step 2 – Motion correction and resolution enhancement**: We processed the CMR segmentations to apply motion correction and enhance spatial resolution, making them more suitable for shape analysis. To this end we used an openly available latent optimisation framework (Wang et al., 2021) trained on a research cohort of cardiac super‐resolution label maps (spatial resolution 1.25×1.25×,2, mm^3^) from 1200 healthy volunteers (Bai et al., 2015; Savioli et al., 2021). This β‐variational autoencoder (VAE) approach performs both resolution enhancement and motion correction by inferring a manifold of anatomically plausible high‐resolution segmentations. The model then leverages a multiview latent optimisation process to iteratively infer the high‐resolution segmentation corresponding to a low‐resolution, motion‐degraded input segmentation. The process allows up scaling the 1.25×1.25×,8 mm^3^ spatial resolution of a routine CMR segmentation, to the 1.25×1.25×,2 mm^3^ resolution of the training label maps (Savioli et al., 2021). For further details about the pretrained networks, we refer the interested reader to the original work by (Wang et al., 2021). Before we applied the pretrained model to our CMR segmentation dataset, we cropped our low‐resolution short‐axis segmentations with a 88×88×12 voxel mask centred around the centre of mass and reorient the cropped segmentation via affine transformations to ensure orientation consistency between the original training and our input dataset. We applied the β‐VAE model to obtain up sampled labels for the RV blood pool, LV blood pool and myocardium. Because of the β‐VAE generative nature, extrapolation can occur where ventricular outflow tracts or other structures are not consistently visible across all subjects; thus we defined a region of interest to standardise the subsequent analysis.

**Step 3 – Region of interest and RV epicardium estimation**: We refined the output segmentations from the β‐VAE model using atrial landmarks, that is, the mitral and tricuspid valve planes, derived from the long‐axis atrial blood pool segmentations (Bai et al., 2018). Each landmark was used to define a cutting plane that is approximately orthogonal to the short‐axis view. The region of interest was defined as the voxels from LV and RV that fall below the mitral and tricuspid planes, respectively. The construction of these planes is detailed in Appendix A. Given that the RV epicardium is difficult to differentiate from the RV endocardium in clinical CMR images, we generated an RV myocardium by offsetting the RV blood pool label by a specified thickness of 3 mm using binary dilation (Schuler & Loewe, 2021). We calculated the dilation radius based on the spacing of the voxels, excluding the upper slice of the RV blood pool to keep the newly generated RV myocardium open. Finally we merged the LV myocardium label with the generated RV myocardium label to form a unified biventricular myocardium label as shown in Fig. 2*C*.

#### Mesh extraction and alignment

Following segmentation preprocessing, we generated a surface mesh of the biventricular myocardium, which provides a connected and spatially coherent representation of the cardiac anatomy. We first applied Taubin smoothing (Fedorov et al., 2012; Valette et al., 2008), which preserves edges and interfaces within segmented structures. This choice maintained the necessary structural details and anatomical integrity while reducing noise and irregularities. Subsequently we extracted a surface mesh from the label using the flying edges algorithm (Schroeder et al., 2015). Flying edges is an isocontouring algorithm which approximates an isosurface from a three‐dimensional discrete scalar field by subdividing the field into uniform cubes and processing only the edges that intersect the isosurface. We uniformly remeshed the resulting surface meshes from all subjects using Voronoi clustering (Valette & Chassery, 2004; Valette et al., 2008), targeting 20,000 nodes per mesh in line with similar shape modelling application in the cardiovascular field (Bai et al., 2015; Cutugno et al., 2021). As a result we obtained $N_{P}=456$ surface meshes with an equal number of vertices. Given the natural biventricular positioning and orientation variability in the thorax, and as such the global coordinate system of the CMR images, the resulting meshes still retained differences in alignment. As we were solely interested in cardiac morphological variability, we performed an alignment step to eliminate these positional differences. We aligned the cohort meshes using the iterative closest point (ICP) algorithm as implemented in the Visualization Toolkit package (Schroeder et al., 2006; Zhang, 1994). ICP is a widely used alignment method that finds the optimal rigid transformation that minimises the distance between corresponding points in the meshes, where correspondence is based on spatial proximity. Further details about the ICP algorithm are provided in Appendix B. Our alignment proceeded in two steps. We first rigidly pre‐aligned each mesh to a randomly selected reference mesh within the cohort. Next all surface meshes were aligned to a generic biventricular mesh, used as reference. By pre‐aligning the meshes, we reduced global transformation differences and improved the reliability and convergence of the final alignment. The alignment to the generic biventricular template allowed preparing the cohort for the anatomical mapping process and avoided bias in reference selection.

### Cardiac atlas construction

Before dimensionality reduction we needed to establish point‐wise correspondence across the biventricular anatomies of all subjects to yield meaningful modes of shape variation. To achieve this purpose we used large deformation diffeomorphic metric mapping (LDDMM) to map the extracted biventricular meshes to a generic biventricular template, followed by principal component analysis (PCA) to construct the cardiac atlas.

#### Large deformation diffeomorphic metric mapping

**Figure 3:**
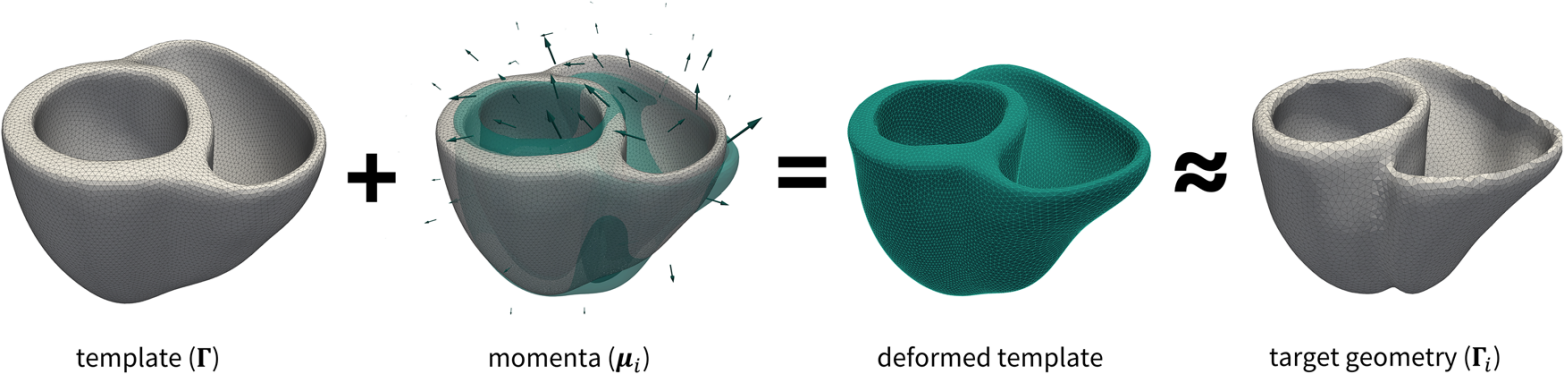
Overview of the large deformation diffeomorphic metric anatomical mapping framework The framework optimises the template geometry to match the target anatomy through the application of momenta at the control points. These momenta drive the deformation of the template mesh, iteratively minimising the vertex‐wise distances between the deformed template and the target geometry. The final set of optimised momenta produces a deformation that closely approximates the target geometry.

LDDMM is a mathematical framework for analysing and comparing shapes, images and structures via smooth, invertible transformations, called diffeomorphisms (Glaunès et al., 2008). This framework deforms a template geometry towards target geometries using diffeomorphisms $\Phi_{i}:R^{3}\mapsto R^{3}$ defined on an ambient space $R^{3}$. These diffeomorphisms are fully parameterised by a predefined grid of $N_{q}$ equally spaced control points ${\left(q_{n}\right)}_{n=1,\cdots ,N_{q}}$ and a to‐be‐optimised corresponding set of momenta ${\left(\mu_{n}\right)}_{n=1,\cdots ,N_{q}}$. Figure 3 showcases how these pairs of control points and momenta deform the template geometry through the ambient space. More specifically the action of the subject‐specific $\Phi_{i}$ on the template surface mesh Γ approximates the target surface mesh $\Gamma_{i}$ as follows:

(1) $$\Gamma_{i}\approx \Phi_{i}\left(\Gamma \right)=\Phi_{q,\mu_{i}}\left(\Gamma \right).$$

Following the details on the construction of these diffeomorphisms provided in Appendix C, the final template geometry Γ and momenta μ are found by minimising the following cost function:

(2) $$\begin{array}{ccc} L\left(\Gamma ,\mu \right) & = & \sum_{i=1}^{N_{P}}\frac{1}{\sigma^{2}}d_{W}{\left(\Phi_{q,\mu_{i}}\left(\Gamma \right),\Gamma_{i}\right)}^{2}\\ & & +\;\sum_{i=1}^{N_{P}}\mu_{i}^{⊤}K_{W}\left(q,q\right)\mu_{i}, \end{array}$$

where $N_{P}$ is the number of target geometries. The first term contains the sum of the Varifold distances $d_{W}$ ‐ eqn (A.10) ‐ which represents the vector distances between the target geometries $\Gamma_{i}$ and their approximations through $\Phi_{q,\mu_{i}}$. The second term penalises deformations with high kinetic energy $K_{W}$ ‐ eqn (A.9), and σ scales the relative importance between the two terms. To optimise eqn (2), two steps are iterated until convergence (Bône et al., 2018): (i) Optimisation with respect to the momenta μ while keeping the template Γ fixed, yielding the $N_{P}$ deformations that map the (current) template to each target geometry. (ii) Optimisation with respect to the template Γ while keeping the momenta μ fixed, resulting in an updated template derived from the (current) set of subject‐specific momenta. The first step computes the subject‐specific deformations needed to match the template to each target anatomy, establishing point‐to‐point correspondence between the template and the target mesh. The second step ensures that the final template reflects a population‐wide anatomical baseline, facilitating both physiological interpretability and more accurate shape modelling. For further details about the LDDMM framework and the optimisation approach, we refer the interested reader to the original work (Bône et al., 2018; Durrleman et al., 2014). Once optimised, the cohort of geometries is represented by the final template Γ and a unique set of momenta $\mu_{i}$ for each subject, that is, target geometry i. Applying these momenta to the template vertices reconstructs the corresponding biventricular surface meshes for each subject, ensuring consistent point‐to‐point correspondence across all meshes in the cohort. In practice this process entails the selection of an initial template $\Gamma_{0}$ (see Section 2.2.2), to which the surface meshes need to be aligned, and two user‐defined hyperparameters $\lambda_{V}$ and $\lambda_{W}$ (see Section 2.2.3). We applied this framework to our cohort of biventricular meshes, performing hyperparameter optimisation to identify the optimal parameters, and used these to anatomically map all geometries to a generic biventricular template.

#### Generating the biventricular template

To generate an initial generic biventricular template $\Gamma_{0}$, we adapted the generic human heart scaffold from the SPARC project (Osanlouy et al., 2024; Soltani et al., 2024). This scaffold, originally a detailed 3D volumetric model with separately annotated structures (e.g. ventricle walls, atrial appendages, inlets), was pruned to focus solely on the biventricular anatomy. Specifically we removed the luminal and outer surfaces of the right and left atrium, as well as the inferior and superior vena cava outflow tracks, and performed a manual cutting operation similar to Section 2.1.2, to remove the LV and RV outflow tracts. Finally we remeshed the remaining biventricular structure using the Voronoi clustering method from Section 2.1.3. By providing a similar structure in the template and target meshes, we enhanced the Varifold distance calculation and improved the robustness of the optimisation process during mapping.

#### Anatomical mapping

We used the LDDMM framework (Section 2.2.1) implemented in *Deformetrica* (Bône et al., 2018) to anatomically map the entire cohort of $N_{P}$ surface meshes (Section 2.1.3). This process involved setting two hyperparameters from the LDDMM framework: $\lambda_{V}$, the kernel width of $K_{V}$ in eqn A.7 – also known as the *stiffness* parameter – that promotes more global deformation, and $\lambda_{W}$, the kernel width of $K_{W}$ in eqn (A.9) – the *resolution* parameter which promotes capturing higher level of details (Bruse et al., 2016). We optimised these hyperparameters by evaluating the reconstruction error between each original mesh and its reconstructed counterpart, that is, obtained by applying the optimised subject‐specific deformation to the template. For subject i, let ${\left(p\right)}_{j=1,\cdots ,N_{v}}$ be the set of vertices in the original mesh, and ${\left(r\right)}_{j=1,\cdots ,N_{v}}$ be the corresponding vertices in the reconstructed mesh, where $N_{v}$ is the total number of points. We calculated the reconstruction error $\ell_{i}$ for subject i as:

(3) $$\ell_{i}=\frac{1}{N_{v}}\sum_{j=1}^{N_{v}}∥p{\left(j\right)}_{i}-r{\left(j\right)}_{i}∥,$$

where ${∥p\left(j\right)}_{i}-r{\left(j\right)}_{i}∥$ is the Euclidean distance between the j‐th vertex point on the original mesh and the j‐th vertex point on the reconstructed mesh for subject i. The total reconstruction error $\ell_{\text{tot}}$ for the cohort of $N_{P}$ subjects is then given by:

(4) $$\ell_{\text{tot}}=\frac{1}{N_{P}}\sum_{i=1}^{N_{P}}\ell_{i}.$$

We varied both $\lambda_{V}$ and $\lambda_{W}$ from 4 to 20 mm in increments of 2 mm. To reduce computational costs, we focused on mapping the five most extreme anatomies in the cohort, identified by their distances from the template. This approach, following similar methodologies (Bruse et al., 2016; Verstraeten et al., 2024), assumes that accurately capturing the most challenging shapes sets a lower bound on deformation *stiffness* and *resolution* to obtain an appropriate matching. Of the top 10 hyperparameter combinations evaluated, we qualitatively based our choice on the following criteria: (i) reconstruction accuracy, measured via the total reconstruction error; (ii) anatomical plausibility, assessed by visual inspection to detect overfitting or local unrealistic warping; and (iii) acceptable computational runtime. The trade‐off between these criteria led to $\lambda_{V}=10$, mm and $\lambda_{W}=6$, mm. Using these optimised hyperparameters, we anatomically mapped the entire biventricular cohort to the derived generic template, producing a new cohort of $N_{P}$ surface meshes with consistent point correspondence. To ensure a correct anatomical mapping, we assessed the total reconstruction error for the full cohort of $N_{P}$ meshes. Finally we performed dimensionality reduction on the mapped meshes to characterise the cohort's cardiac morphological variability.

### Statistical shape modelling via dimensionality reduction

We applied PCA to the mapped cardiac meshes to retrieve a compact representation of the anatomical variability in our studied cohort and a set of low‐dimensional shape descriptors that could be subsequently used to quantify and compare morphological patterns across individuals. PCA projects high‐dimensional data onto a linear subspace aligned with the directions of maximum variance, significantly reducing the number of retained variables while preserving most of the variability in the original data. In our statistical shape analysis case, we applied PCA to the vertex coordinates of the aligned surface meshes in the cohort which we ensured to be in correspondence. The resulting decomposition provided an updated average template and a set of complex shape modes that represent the modes of anatomical variation. These features could then be used to morphologically characterise each subject in the cohort. Mathematically PCA involves the eigen‐decomposition of the covariance matrix of dimensions $3\times N_{v}\times N_{P}$, where $N_{v}$ is the number of vertices per mesh and $N_{P}$ is the total number of subjects. The resulting eigen‐decomposition yields eigenvectors ϕ and eigenvalues λ, representing the principal components and the variance explained by each, respectively. By ranking eigenvalues from largest to smallest, we ensured that the first few orthogonal components capture the dominant modes of anatomical variation. Any mesh in the cohort could be reconstructed as:

(5) $$\Gamma_{i}=\bar{\Gamma }+\sum_{m=1}^{N_{M}}a_{i,m}\phi_{m}\lambda_{m},\;i\in \left\{1,2,\text{…},N_{P}\right\},$$

where $\phi_{m}$ is the m‐th principal component, $\alpha_{i,m}$ is the m‐th weight (or *shape coefficient*) for the i‐th subject, $N_{M}$ is the number of principal components used for the reconstruction and $\bar{\Gamma }$ is the average template. These scalar‐shape coefficients quantified each principal component's contribution to an individual's cardiac morphology, effectively characterising each subject through their ensemble of shape coefficients $\alpha_{i,m}$. By retaining principal components that collectively explain a significant portion of the variance, we reduced the dimensionality of anatomical variability. Specifically we retained the first $n<N_{M}$ modes accounting for 90% of the total variance, assuming that the remaining modes primarily capture noise or insignificant variability. To interpret these modes, we deformed the average template along each eigenvector by varying the shape coefficient $\alpha_{m}$ within its respective ± 3 standard deviations range. These synthetic representations empirically illustrate the anatomical variations associated with each mode, providing insights into the dominant patterns of morphological variability in the cohort. Furthermore we performed statistical analysis on the collection of the first n shape coefficients, for each subject in the cohort.

### Statistical analysis – Isolating the sex component

#### Sex‐specific analysis of cardiac shape coefficient distribution

To assess whether the overall cardiac shape differs systematically between males and females, we conducted a sex‐specific analysis comparing the distributions of shape coefficients. We employed Hotelling's T^2^ test to determine whether the multivariate distributions of *sex‐stratified* shape coefficients differ significantly between the male and female subgroups. As the multivariate extension of the Student's *t*‐test, the Hotelling's T^2^ test evaluates whether the mean vectors of two groups are significantly different across multiple variables. The null hypothesis states that the two groups have the same multivariate mean vector. We calculate the T^2^ statistic and the associated *P*‐value, quantifying how far apart the group means are and whether the difference is statically significant. We conducted the Hotelling's T^2^ test both before and after correcting for confounders to evaluate whether sex‐specific differences in the shape coefficients remain significant. In all analyses equality of variance between the groups was assessed through Levene's test and a *P*‐value below 0.05 was considered statistically significant.

#### Multivariate analysis of covariance of cardiac shape coefficients

To investigate how cardiac shape variation is influenced by sex and other potential confounders, we employed multivariate analysis of covariance (MANCOVA). MANCOVA extends the analysis of covariance and regression by assessing multiple dependent variables against both categorical and continuous independent variables. These independent variables, also referred to as *explanatory variables* or *predictors*, are thought to explain the variance in the dependent variables. MANCOVA compares the variance explained by the model to the residual unexplained variance, testing the null hypothesis that predictors have no effect on the dependent variables (Riffenburgh, 2006). We used Pillai's trace as the target metric for our analysis, which we explain in more detail in Appendix D. Using the *statsmodels* package (Seabold & Perktold, 2010), we applied MANCOVA to evaluate the effects of age, systolic blood pressure and various body size measurements on the first *n* shape coefficients. We performed four separate MANCOVA tests, each investigating the effect of sex, age, systolic blood pressure and a different respective body size metric as a predictor: body mass index (*BMI‐model*), body surface area (*BSA‐mode*l), height (*height‐model*) and weight (*weight‐model*). For each model, we evaluated Pillai's trace. Normality was assessed through Kolmogorov–Smirnov test, and a *P*‐value below 0.05 is considered statistically significant for the results of the analysis.

#### Correcting shape coefficients for confounders

To isolate the effects of sex on cardiac shape from those of other influencing variables, we performed a correction step to remove the impact of significant confounders from the shape coefficients. After identifying significant confounders, we used multivariate linear regression to correct the shape coefficients for these variables, excluding sex. Following a similar approach to (Bernardino et al., 2020), we let M represent the set of significant confounding variables. Assuming M to be these confounding variables, we modelled each shape coefficient α as:

(6) $$\alpha =\alpha_{M}+\alpha_{I}+\varepsilon ,$$

where $\alpha_{M}$ captures the shape mode variability due to confounders, and $\alpha_{I}$ represents the variability of other sources, including sex. Our primary interest was in obtaining the corrected shape mode coefficients after removing the influence of the confounders. Thus, we aimed to estimate:

(7) $$\alpha_{corr}=\alpha -\alpha_{M}.$$

Assuming linear contributions of the confounders to $\alpha_{M}$, the corrected shape coefficient becomes:

(8) $$\alpha_{corr}=\alpha -w_{bs}\cdot x_{bs}-w_{a}\cdot x_{age}-w_{bp}\cdot x_{bp},$$

where $w_{bs}$, $w_{age}$ and $w_{bp}$ are the weights corresponding to body size, age and systolic blood pressure, respectively. We used ordinary least squares regression (Seabold & Perktold, 2010) to estimate these weights for the first n shape modes across four different models, each accounting for a different body size metric (BMI, BSA, height, weight). While the weights could be visualized and interpreted, we used them to obtain corrected shape coefficients, denoted as $\alpha_{corr}$ for each model, which served as inputs for our logistic regression analysis.

#### Logistic regression analysis

To evaluate the discriminative power of shape coefficients and assess the impact of confounder correction on sex classification, we performed logistic regression analysis. We trained multiple logistic regression models to determine how well corrected and uncorrected shape coefficients can differentiate between male and female subjects. This analysis estimates the probability that a given subject belongs to a particular class (male or female), based on the values of the independent variables (corrected and uncorrected shape coefficients). First we trained a logistic regression model using the uncorrected shape coefficients as independent variables:

(9) $$\begin{array}{ccc} \mathrm{logit}(\pi (Y=1)) & = & \beta_{0}+\beta_{1}\cdot \alpha_{1}+\beta_{2}\cdot \alpha_{2}\\ & & +\;\cdots +\beta_{n}\cdot \alpha_{n}. \end{array}$$

Here π(Y=1) represents the probability of a subject being male, with Y=1 coded for males and Y=0 for females. $\beta_{0}$ serves as the intercept term and the coefficients $\beta_{1},\cdots ,\beta_{n}$ quantify the relationship between each shape coefficient $\alpha_{1},\cdots ,\alpha_{n}$ and the likelihood of being male. This allowed us to establish a baseline of the shape‐based discrimination between male and female subgroups, without additional correction. Subsequently we trained four logistic regression models using shape coefficients corrected for age, systolic blood pressure and different body size metrics (BMI, BSA, height, weight):

(10) $$\begin{array}{ccc} \mathrm{logit}(\pi (Y=1)) & = & \beta_{0}+\beta_{1}\cdot \alpha_{corr,1}+\beta_{2}\cdot \alpha_{corr,2}\\ & & +\;\cdots +\beta_{n}\cdot \alpha_{corr,n}, \end{array}$$

where $\alpha_{corr,i}$ denote the BMI‐, BSA‐, height‐ or weight‐corrected shape coefficients. To evaluate the goodness of fit for these models, we visualised model performance using receiver operating characteristic (ROC) curves, which represent the trade‐off between the true positive rate (sensitivity) and the false positive rate (1 – specificity) across different classification thresholds. The area under the curve (AUC) serves as a summary measure of the model's ability to correctly classify male and female subjects based on the shape coefficients, reflecting the overall discriminative power of shape coefficients towards sex classification. Finally we analysed the logistic regression coefficients to identify which anatomical variation modes significantly contribute to differentiating between male and female subgroups, using a *P*‐value threshold of 0.05 for statistical significance.

## Results

### Biventricular cardiac atlas

**Figure 4:**
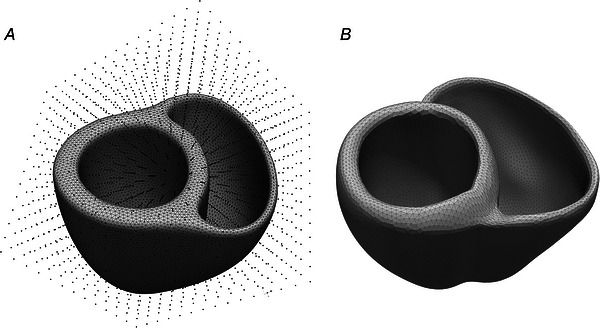
Anatomical mapping of the biventricular surface meshes *A*, The initial biventricular template $\Gamma_{0}$ embedded in the ambient space. *B*, Final population template Γ after mapping.

**Figure 5:**
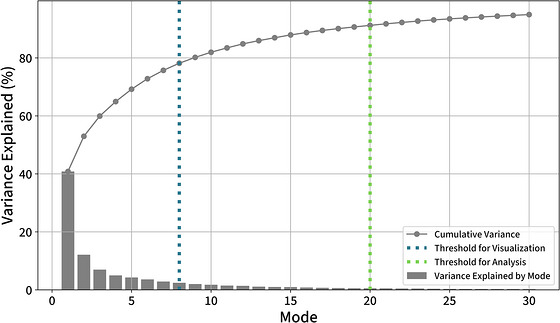
Variance explained by the modes of anatomical variation The bar plot shows the percentage of variance explained by each mode, computed by dividing its eigenvalue by the total sum of eigenvalues. The dotted line represents the cumulative variance explained by the modes. The blue dashed line represents the threshold for the modes selected for visualisation purposes, and the green dashed line represents the threshold for the modes included in the analysis.

Using the framework presented in Section 2.2.1, we use the generic biventricular mesh and the optimised hyperparameters to extract a set of momenta for each of the 456 subjects, together with a final template. The final combination of hyperparameters yields a grid of 990 control points. The initial (idealised) biventricular template, embedded in this ambient space, and the final population‐derived template are shown in Fig. 4. We reconstruct a cohort of $N_{p}=456$ mapped surface meshes by applying each subject‐specific set of momenta to the template, and obtain a total reconstruction error $\ell_{\text{tot}}=0.22$ mm in the entire cohort, with the highest reconstruction error being $\ell_{\max}=0.83$ mm. These reconstruction results are in line with similar applications in cardiovascular research, using both LDDMM and other mapping techniques (Bai et al., 2015; Verstraeten et al., 2024). In addition, as the reconstruction error is below the resolution of the label maps used for mesh extraction, we consider our approximation acceptable. Consequently, we use the cohort of reconstructed mapped surface meshes to train the statistical shape model. We apply PCA on the mapped cohort to obtain the principal components of anatomical variance, as illustrated in Fig. 5. The first n=20 shape coefficients and modes account for 90% of the cohort's anatomical variability, providing a robust foundation to represent and analyse cardiac morphology in our subsequent statistical analyses.

**Figure 6:**
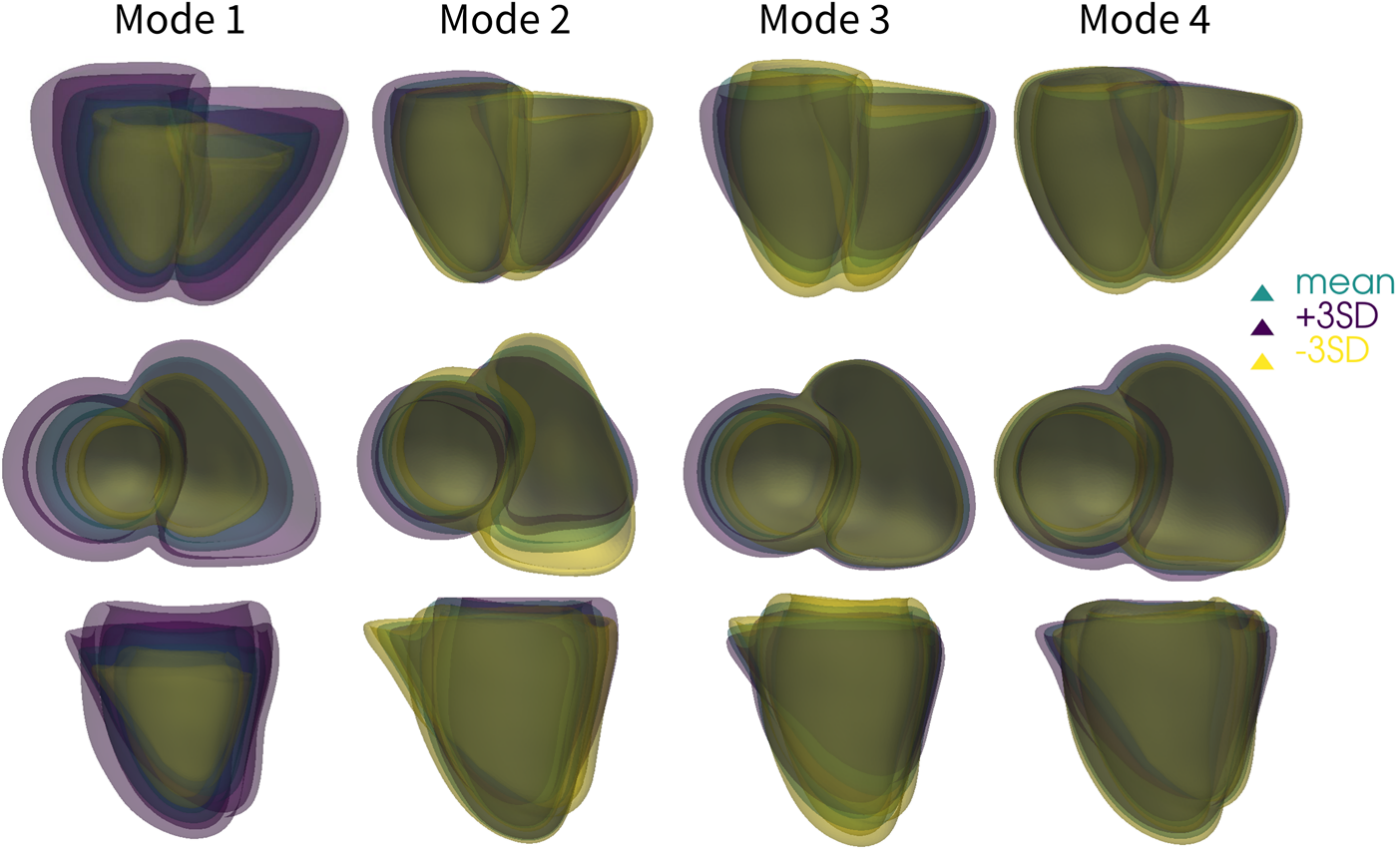
The first four modes of anatomical variation of the biventricular cardiac anatomy in the cohort For each mode, we display overlay of the mean shape and the shape at the ±3 standard deviation (SD).

Figure 6 shows a graphical overview of the first four modes of anatomical variation in our cohort. Animations of the first eight modes, together accounting for about 80% of the variability, can be seen in the supplementary material. Mode 1 accounts for 44.8% of the anatomical variability and is mainly associated with the size of the chambers. Mode 2 accounts for 13.3% of the variability and reflects the variation in the anterior–posterior width of the RV and septum. Mode 3 explains 7.6% of the variability and is related to the longitudinal length of the cardiac chambers. Mode 4 accounts for 5.5% of the variability and captures the relative orientation of the RV respective to the LV. Mode 5 accounts for 4.7% of the variability and is visually associated with the obliqueness of the RV. Mode 6 accounts for 4.0% of the variability and is linked with the bulging of the LV. Mode 7 accounts for 3.2% of the variability and is visually associated with the sphericity of the RV. Mode 8 accounts for 2.7% of the variability, and from visual association can be related with the morphology of the RV free wall.We use the shape coefficients from the first n=20 modes for each subject to represent and analyse cardiac morphology in the statistical analysis.

### Uncorrected sex‐stratified shape coefficients distribution

**Figure 7:**
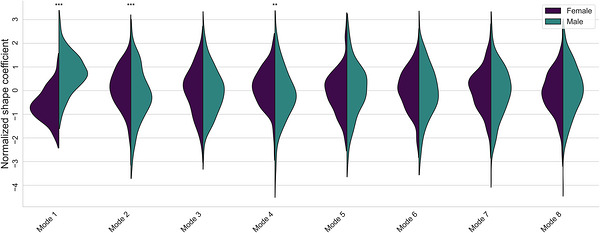
Distribution of the shape coefficients for the first eight modes, stratified by sex Purple represents females, and teal represents males. The shape coefficients are provided as *z*‐scores normalised by their mean and standard deviation. Asterisks above the violins indicate statistical significance of the difference between male and female distributions based on an independent *t*‐test: * indicates P<0.05, ** indicates P<0.01, *** indicates P<0.001.

The violin plots in Fig. 7 show the distribution of the first eight shape coefficients, stratified by sex. Upon visual inspection, we observe that modes 1 and 2 show significant differences in the mean shape distributions between males and females. Considering the visual interpretation of these modes from Fig. 6, these differences suggest significant sex‐specific variation in mean overall chamber size (Mode 1), as well as the transverse width of the RV and septum (Mode 2). Mode 4 also shows a statistically significant difference, suggesting sex‐specific differences in the relative orientation of the RV respective to the LV, although less pronounced than the first two modes. While Mode 5 also exhibits noticeable visual differences in both means and variances, the difference is not statistically significant, although it might reflect heterogeneity in RV obliqueness, particularly among males. Modes 6 and 7 again reveal subtler variations, particularly in the tails of their distributions and mean values, suggesting that males present more extreme anatomical variations in terms of LV bulging (Mode 6) and RV sphericity (Mode 7). However, these differences do not appear to be statistically significant based on the independent *t*‐test result. Furthermore, with respect to the multivariate distributions of the shape coefficients, the Hotelling T^2^ test indicates a highly significant statistical difference between the two distributions, that is, P<<0.001 and $T^{2}=585.32$ (Table 2). This result confirms significant differences in cardiac morphology between the male and female biventricular anatomies.

**Table 2:** Hotelling's test on the uncorrected and corrected shape coefficients. The T^2^ statistic measures the distance between the means of the two groups, quantifying how far apart the group means are, adjusted for the data's covariance structure. Corresponding F‐ statistics and *P*‐values confirm statistical significance.

	Hotelling's T^2^	F‐statistic	*P*‐value
Uncorrected model	585.32	28.03	1.63e‐65
BMI‐corrected model	455.50	21.82	2.12e‐53
BSA‐corrected model	76.24	3.65	2.77e‐07
Height‐corrected model	73.60	3.52	6.32e‐07
Weight‐corrected model	131.33	6.29	6.32e‐15

### Effect of confounders on cardiac morphology

**Figure 8:**
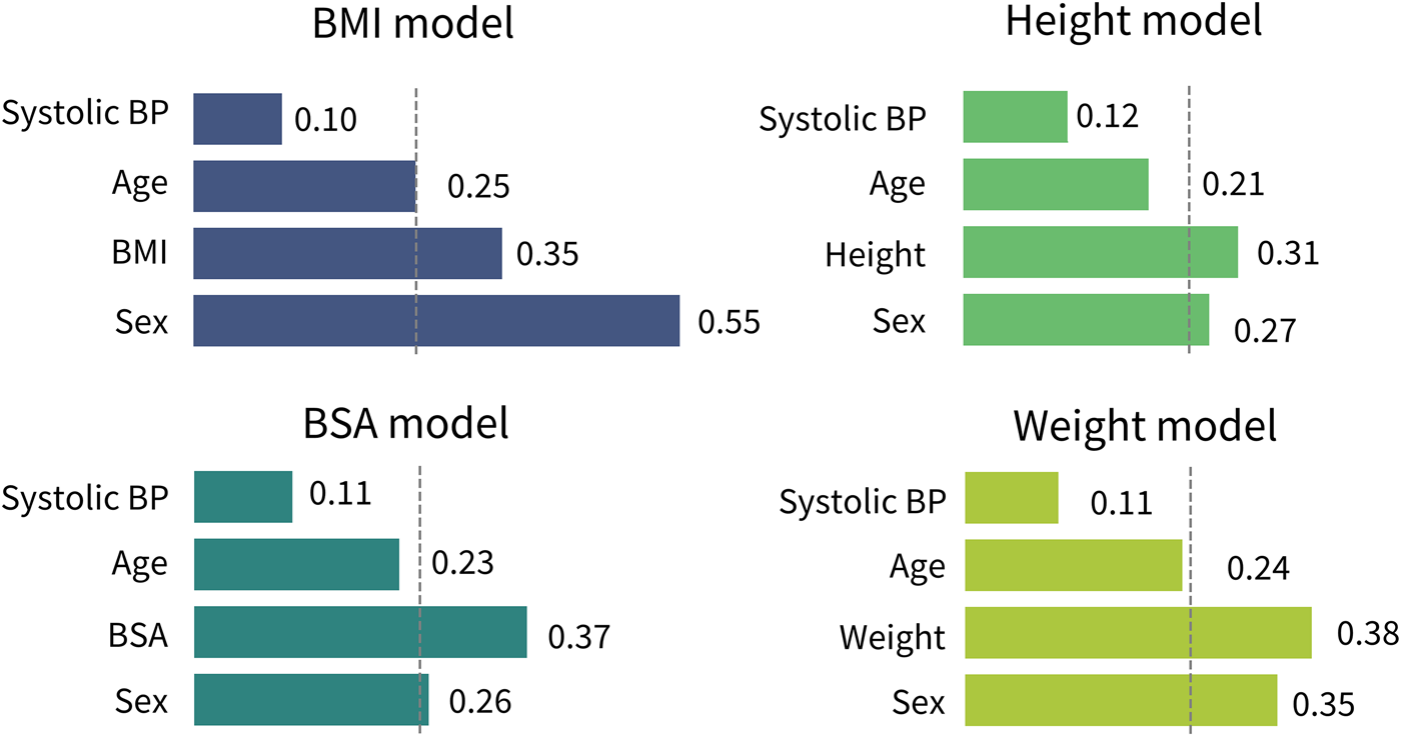
Contribution of explanatory variables to cardiac shape variability among the four different models Each bar represents Pillai's trace, which quantifies the amount of variance in the shape coefficients attributed by each explanatory variable after correction for the other confounders. It is calculated as the sum over all predictors p, of the fraction $\lambda_{p}/\left(1+\lambda_{p}\right)$, where $\lambda_{p}$ are the eigenvalues of the matrix $A=HE^{-1}$, with H and E being the model sum of squares and residual sum of squares matrices, respectively. A value of 0 indicates no contribution of the explanatory variable to cardiac shape variability, while a value closer to 1 suggests a strong effect. All explanatory variables have a statistically significant influence on morphological variability, and sex explains at least 25% of the variability in all models.

From the MANCOVA conducted on the first 20 shape coefficients, we observe that all explanatory variables, including BMI, BSA, height, weight, age and systolic blood pressure, significantly affect overall cardiac morphology, with P≪0.001 across all variables and models. However their contributions to explaining cardiac morphological variance, quantified by Pillai's trace (Fig. 8), are generally smaller than that of sex. Specifically the variance in the shape coefficients explained by sex, after accounting for other confounders, ranges from 0.25 in the height‐model to 0.55 in the BMI‐model, demonstrating that sex consistently accounts for a substantial portion of cardiac morphological variability.

The results for the individual models are summarised as follows:

- BMI‐corrected model: Sex explains 54.6% of the cardiac anatomical variance, which is the largest sex‐based shape contribution among all models. This suggests that, even when adjusting for BMI, sex strongly influences cardiac morphology.
- BSA‐corrected model: After accounting for BSA, sex still accounts for 26.1% of the cardiac morphological variability, showing a decrease in explained variability relative to the BMI correction, yet remaining a dominant factor.
- Height‐corrected model: After accounting for height, sex accounts for 30.5% of the cardiac anatomical variance, showing a very similar behaviour as the BSA‐corrected model.
- Weight‐corrected model: After accounting for weight, sex still accounts for 34.7% of the cardiac morphological variability. Here sex accounts for less shape variability than the BMI‐corrected model but still more shape variability than the BSA‐ and height‐corrected models.

From this analysis we observe that although adjustments for anthropometric covariates slightly reduce the proportion of morphological variance explained by sex, it remains consistently high throughout all correction models.

Table 2 reports the Hotelling's T^2^ test results for the confounder‐corrected shape coefficients, that is corrected with respect to age, systolic blood pressure and the selected body size metric. In all models the *P*‐value is well below the significance threshold, indicating a statistically significant difference in the distribution of the coefficients between the male and female subgroups, for all the corrected coefficients. The T^2^ statistics reveal that corrections using age, systolic blood pressure and height (height‐corrected model) result in the smallest differences in the mean shape coefficients between the two groups, whereas the BMI‐model leads to differences closer to the uncorrected model. In addition, BSA‐corrected shape coefficients follow a trend similar to that of the height‐corrected coefficients, while a combined age, systolic blood pressure and weight correction (weight‐corrected model) yields less pronounced differences in the means compared to BSA‐ and height‐corrected shapes, yet still considerably lower than those seen in the uncorrected and BMI‐corrected models. These results imply that statistically significant sex differences are present in the multivariate collection of morphological descriptions, regardless of the correction for confounders.

### Correlation of cardiac shape and sex

**Figure 9:**
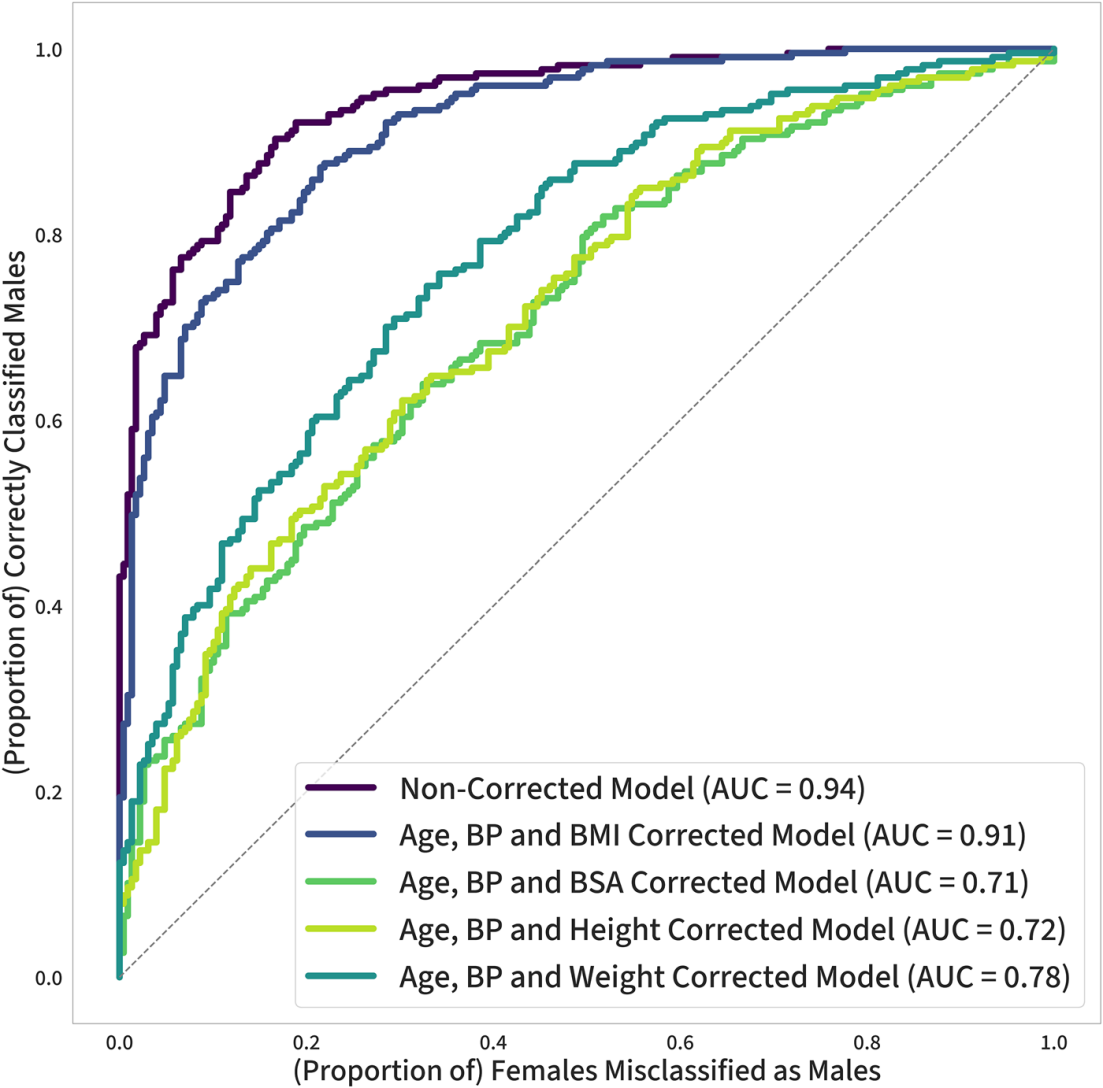
Receiving operator curves (ROC) of the trained logistic regression models, with their corresponding areas under the curve (AUC) The uncorrected model shows the highest discriminative power, achieving an AUC of 0.94. Correction for age, blood pressure (BP) and BMI leads to a slight reduction in AUC to 0.91, suggesting that while BMI accounts for some morphological differences, significant sex‐based distinctions persist. Further corrections for BSA, height and weight – next to age and blood pressure – result in greater reductions in AUC (0.71–0.78). Despite these decreases, all models remain above the discrimination threshold (dotted line), confirming that cardiac morphology retains intrinsic sex differences even after accounting for confounders.

We train logistic regression models using both uncorrected and corrected shape coefficients to evaluate the power of cardiac morphology to discriminate sex before and after adjusting for confounders. Figure 9 presents the ROC curves and corresponding AUC values, which measure each model's ability to discriminate between sexes. Further details about pseudo‐*R* ^*2*^ and the log‐likelihood ratio (LLR) *P*‐values of the trained models are provided in Appendix E. The uncorrected model exhibits strong discriminatory power with an AUC of 0.94, providing strong evidence of pronounced sex dimorphism in cardiac morphology. Although slightly reduced, the BMI‐corrected model still retains high discriminatory performance, with an AUC of 0.91, indicating that the sex‐based influence on cardiac morphology persists even after adjusting for age, systolic blood pressure and BMI. This suggests that these confounders do not fully explain the morphological distinctions between male and female hearts and are not sufficient for fully correcting sex‐related cardiac morphological variation linked to body size. In contrast, corrections for BSA, height and weight, along with age and blood pressure, lead to a more pronounced drop in discriminatory model performance, with corresponding AUC values of 0.71, 0.72 and 0.78. These reductions illustrate the contribution of body size and scaling factors to observed sex differences and suggest that BSA and height are the most efficient body size measurements for sex‐specific correction. Nevertheless, even in these corrected models, the AUC values remain well above random classification threshold AUC = 0.5, indicating that intrinsic sex differences in cardiac morphology persist beyond the effects of body size, age and blood pressure. To further explore these differences, we analyse the logistic regression coefficients to identify shape features that contribute most significantly to sex‐based morphological variation in cardiac anatomy.

### Sex‐specific discriminatory shape variation patterns

**Figure 10:**
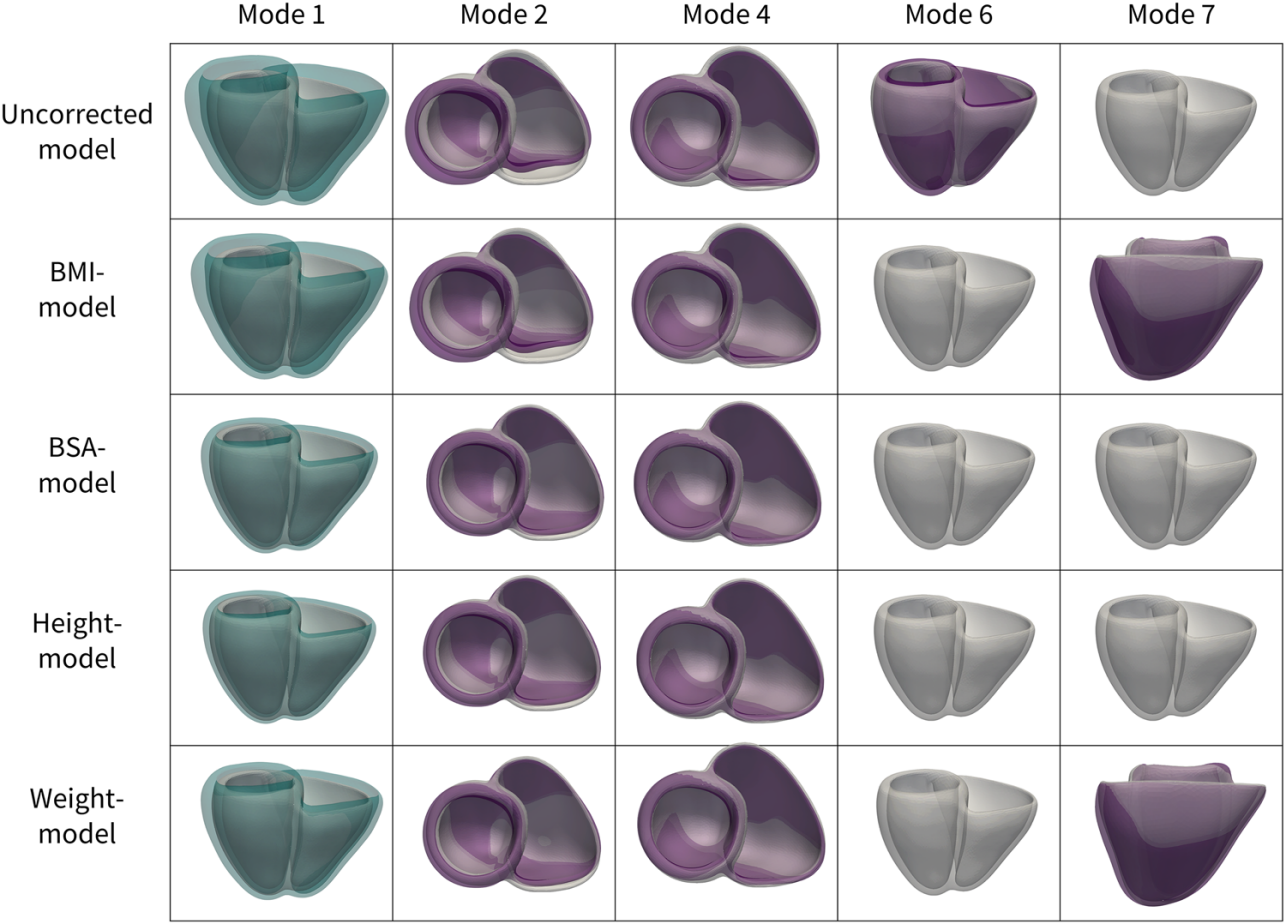
Main morphological discriminators for each model The trends of morphological discrimination between male and female subjects are summarised for each model. Purple shading indicates that the depicted morphological variation distinguishes female against males, while teal shading indicates the opposite. The magnitude of the depicted variation is proportional to the magnitude of the corresponding logistic regression coefficient.

Table 3 reports the logistic regression coefficients for shape modes that significantly contribute to sex‐based discrimination in at least one of the correction models. Here negative coefficients indicate modes that characterise the female group. Figure 10 provides a graphical summary of these morphological trends.

**Table 3:** Logistic regression coefficients for all models. All reported values are statistically significant, while '/' denotes results that were not statistically significant. Negative coefficients indicate a stronger association with the female population, whereas positive coefficients indicate discrimination towards the male population.

Coefficient	Uncorrected	BMI‐corrected	BSA‐corrected	Height‐corrected	Weight‐corrected
Mode 1	3.0272	2.7706	1.1542	0.9481	1.5325
Mode 2	−0.8451	−0.7564	−0.2796	−0.2950	−0.3181
Mode 4	−0.7597	−0.7055	−0.2834	−0.2927	−0.3557
Mode 6	−0.5208	/	/	/	/
Mode 7	/	−0.3502	/	/	−0.2054
Mode 13	−0.4116	−0.3963	/	/	/
Mode 17	−0.4698	−0.4681	−0.2245	/	−0.2937

Mode 1 consistently shows the highest positive coefficients in all models, with values ranging from 3.03 in the uncorrected model to 0.94 in the height‐corrected model. This indicates that Mode 1 is a dominant factor in distinguishing male and female subjects, although its influence diminishes after accounting for age, systolic blood pressure and body size measures. The visual interpretation of Mode 1 suggests that heart size is the strongest differentiating feature, with larger hearts being characteristic of males. Mode 2 and Mode 4 demonstrate moderate negative coefficients across all models, with values ranging from –0.86 to –0.28 for Mode 2 and –0.76 to –0.28 for Mode 4 in the uncorrected and BSA‐corrected models, respectively. This evidence indicates that both modes moderately contribute to the shape‐based classification of female versus male hearts regardless of the applied corrections, although their effects diminish after accounting for confounders, and are less prominent compared to Mode 1. The visual interpretation of these modes (Fig. 6) indicates that a smaller RV and its relative positioning to the LV are key distinguishing features of female hearts. Mode 6 is characterised by negative coefficient (–0.5208) in the uncorrected model, suggesting an association with female cardiac morphology. However, this mode is not significant in the corrected models, indicating that its discriminatory power is highly sensitive to confounding factors. This may suggest that Mode 6 captures features proportionately influenced by body size, age and blood pressure. Comparing trends in other coefficients, it is possible that Mode 6 is more influenced by age or systolic blood pressure, rather than body size. The visual interpretation of this mode suggests that female hearts exhibit greater LV apex by bulging, which could be linked to age or blood pressure effects. Mode 7, visually associated with the roundness of the RV, is a non‐significant mode for sex discrimination in most correction models except in the BMI‐ and weight‐corrected models, where it has coefficients of −0.3502 and −0.2054, respectively. This indicates that, when adjusting for BMI and weight, Mode 7 has a modest ability to characterise the female cardiac morphology based on the roundness of the RV. Mode 13 consistently displays negative, but relatively minor, coefficients (−0.4116 in the uncorrected and −0.3963 in the BMI‐corrected model) but is not significant in the other corrected models. Similarly Mode 17 shows significant negative coefficients in several models, ranging from −0.4698 in the uncorrected model to −0.2937 in the weight‐corrected model, but not reach significance in the height‐corrected model.

## Discussion

Decades of underrepresentation of women in cardiovascular research have led to a vast knowledge gap in understanding the anatomical, functional and histological differences between male and female hearts (Martin & Leinwand, 2024; Prajapati et al., 2022; St. Pierre et al., 2022). Yet, sex‐based differences, that is, sex dimorphism, in cardiac structure and function are increasingly recognised as critical factors influencing divergent responses to cardiovascular risk factors, as seen in conditions like heart failure (Beale et al., 2018; Ji et al., 2022). Therefore an in‐depth investigation of sex differences in cardiac morphology is essential to unravel sex‐specific pathological trajectories in heart disease. In our study we set up a robust statistical shape analysis pipeline to achieve a complete three‐dimensional morphological characterisation of the biventricular cardiac anatomy from CMR images. Leveraging this data‐driven morphological characterisation, we identify the key morphological features that distinguish the male and female cardiac anatomy, while accounting for the effect of confounding variables such as age, blood pressure and various body size metrics.

**Statistical shape modelling highlights significant sex differences in cardiac morphology**. Statistical shape modelling provides a powerful framework to quantify complex morphometric features that go beyond traditional clinical measurements (Bruse et al., 2016; Sophocleous et al., 2022). Its effectiveness in cardiology has been well demonstrated: a challenge from the Cardiac Atlas Project clearly showed that shape modelling‐derived parameters outperform conventional measurements in characterising myocardial infarction (Suinesiaputra et al., 2018), while recent work on Tetralogy of Fallot patients found that biventricular shape modes offer superior predictive support for pulmonary valve replacement compared to standard image‐based metrics (Govil, Crabb et al., 2023). Despite prior work acknowledging sex as a confounding factor in cardiac morphology, to the best of our knowledge, no dedicated statistical shape analysis explicitly focused on sex differences in cardiac anatomy is currently available in the literature. In this work we address this gap by presenting a statistical shape analysis fully focused on characterising sex‐based differences in cardiac anatomy using rich imaging data‐derived morphological features. Our trained statistical shape model of the biventricular anatomy captures 90% of the morphological variability in the cohort with just 20 shape coefficients per subject. The compactness of the model – defined by the number of modes needed to explain a substantial portion of the variability – and the visual interpretation of its modes are in line with what was previously proposed in the literature, given the population size and anatomical complexity under study (Burns et al., 2024; Bai et al., 2015; Mauger et al., 2019; Rodero et al., 2021). As reported in Table 2, the distribution analysis on the shape coefficients using the Hotelling T^2^ test confirms a highly significant shape difference between the male and female subgroups (P<<0.001, $T^{2}=585.32$). This result indicates a clear separation between the two multivariate distributions, reinforcing the presence of substantial sex differences in cardiac anatomy. In addition, as shown in Fig. 7, differences are present in the means, variance and tails of the derived morphological descriptors. This evidence suggests that sex differences in cardiac anatomy are not limited to average shape but span the entire morphological distribution. This implies that female and male hearts differ not only in typical geometry but also in the range of and variability in possible anatomical configurations, further highlighting the need for sex‐specific reference models in both clinical and research settings (van der Kruk, 2025).

**Sex explains at least 25% of the variability in cardiac morphology**. Multivariate analysis of covariance enables a comprehensive assessment of the effect of different confounding factors on cardiac morphology. Our analysis shows that sex, age, systolic blood pressure and body size are significant explanatory variables for cardiac morphology (Fig. 8). While the variability attributed to body size varies based on the specific body size metric considered (i.e. BMI, BSA, height or weight), sex consistently accounts for at least 25% of the variability – even after accounting for age, systolic blood pressure and body size. This finding aligns with a recent ex vivo morphological analysis on normal hearts, which analysed the dimensions of several cardiac structures, while accounting for sex, age and body measurements (Westaby et al., 2023). The study identified sex as the strongest predictor of cardiac anatomy. Our results reinforce this conclusion through a data‐driven statistical shape analysis, conducted on *in vivo* imaging data. Unlike conventional measurements as diameter, lengths and volumes, our approach captures complex three‐dimensional variations, offering a more nuance and comprehensive characterisation of sex‐based differences in cardiac anatomy. Furthermore recognising the magnitude of this sex effect supports the need for sex‐stratified analyses, and the need to include sex‐based anatomical variability in diagnostic pathways.

**Regression analysis identifies the most discriminant modes of anatomical variation between male and female populations**. In statistical shape modelling and complex morphological characterisation, regression analysis is often used to eliminate confounders from shape or to discriminate healthy and disease individuals based on morphological scores (Bernardino et al., 2020; Cutugno et al., 2021; Govil, Crabb et al., 2023; Sophocleous et al., 2022). A recent study introduced a confounding deflation strategy using dimensionality reduction and logistic regression to isolate cardiac remodelling patterns in endurance athletes (Bernardino et al., 2020). This approach involved correcting shapes within the cohort via partial least squares regression and applying logistic regression to identify the most discriminative morphological pattern between athletes and controls (Bernardino et al., 2020). In a similar fashion we use multivariate regression to correct for confounders in cardiac morphology, albeit applying the correction on the shape coefficients obtained after principal components analysis, instead of correcting the raw shape data. This allows us to systematically analyse and compare the shape coefficients before and after correction. Repeated Hotelling T^2^ test on the corrected shape coefficients confirms that while correction diminishes the separation between the multivariate distribution, significant differences between the male and female groups remain across all correction schemes (Table 2). To further quantify the effect of these corrections, we apply logistic regression on the whole ensemble of shape coefficients, assessing both the discriminative power of cardiac morphology and the impact of different correction methods. This approach not only evaluates the overall shape‐based classification performance between the male and female population but also enables the identification of (multiple) anatomical variation modes that significantly contribute to sex differences, rather than isolating a single discriminative pattern (Fig. 10).

**Sex matters: no confounder correction scheme fully eliminates geometric differences between male and female hearts**. By observing the results of our regression models in Fig. 9 and Table A.1, we observe that cardiac morphology alone is a very strong discriminant for sex. This finding aligns with our repeated Hotelling's T^2^ tests, which consistently highlight significant differences in cardiac morphology between the male and female population. Our logistic regression model, trained on the uncorrected shape coefficients, achieves an AUC of 0.94, demonstrating that overall cardiac morphology can discern males against females with a very high sensitivity and specificity. Examining the models trained on corrected coefficients, we observe that while correction for confounders decreases the discriminative power, none of the models approaches the random classification threshold (AUC = 0.5). This confirms the existence of important, intrinsic morphological features that discriminate male and female cardiac anatomies that persist even after accounting for body size, age and blood pressure. These results align with the recent claim advanced that allometric scaling alone is not sufficient to fully eliminate sex differences in cardiac anatomy (St. Pierre et al., 2022). In a broader sense the persistence of cardiac morphology discrimination underscores the presence of fundamental anatomical differences that extend beyond artefacts of confounding variables. These differences are likely rooted in genetic or developmental factors inherent to each sex, rather than being mere consequences of body size disparities (Burns et al., 2024; Lin et al., 2023; Martin & Leinwand, 2024; Reue & Wiese, 2022; Winham et al., 2015). Nonetheless accounting for these intrinsic differences in clinical biomarkers and imaging‐based diagnostic thresholds (Shiwani et al., 2025) will be essential to reduce sex‐related disparities in cardiovascular care.

**Mode 1, Mode 2 and Mode 4 are the most discriminative shape patterns between the male and female population populations, consistently contributing to the separation in all models**. As shown in Table 3, Mode 1, Mode 2 and Mode 4 consistently emerge as the most significant contribution to sex‐based differences in cardiac morphology, maintaining their statistically significant power across all models. Relating these findings to their visual interpretations (Figs. 6 and 10), we identify key morphological distinctions: female hearts are characterised by smaller cardiac chambers, a reduced anterior–posterior width of the RV, and differences in the relative orientation of the RV respective to the LV – differences that persist even after correction for age, body size and systolic blood pressure. While according to our analysis cardiac size is the strongest discriminator between male and female heart anatomies, cardiac differences go beyond just differences in size, and are persisting after correction for confounders. These observed patterns align with reported residual differences in chamber size even after scaling by lean body mass (St. Pierre et al., 2022), suggesting that the cardiac chambers do not scale proportionally between sexes. The discriminative patterns we observe also align with a recent study that used statistical shape analysis to investigate the genetic basis of cardiac morphology (Burns et al., 2024). Similar to our findings, the study reports a strong correlation between principal component 1 (size) and male sex, as well as a correlation between principal component 3 and female sex, indicating that women present smaller anterior–posterior width. In addition, a correlation between sex and RV orientation is also reported, which agrees with our observation on Mode 4 being a sex‐specific discriminant of cardiac morphology. Interestingly the same study reported a correlation between principal component 2 and female sex, suggesting that women have less elongated ventricles. While our visual interpretation of Mode 3 captures variations in ventricular longitudinal length, we do not observe a significant contribution of this mode to sex‐based differentiation. Notably the aforementioned analysis included more than 45,000 subjects from the UK Biobank population without health‐based selection criteria. As a result the observed sex‐specific trend in ventricular elongation may either reflect disease‐related remodelling rather than inherent differences in healthy cardiac anatomy, or result from differing sample sizes between the studies.

**Limitations**. While this study presents a novel statistical shape analysis to investigate sex differences in cardiac anatomy, several limitations should be acknowledged. First we selected a reasonably large cohort of healthy subjects from the UK Biobank population, enabling us to focus on baseline anatomical sex differences. However our analysis was restricted to individuals of Caucasian ethnicity, which may limit the generalisability of our results to other ethnic groups. Extending the analysis to a larger and more diverse population that includes different ethnic backgrounds would enhance the applicability of our findings. Second, although we corrected for key demographic and physiological confounders (e.g. age, body size, systolic blood pressure), unmeasured variables such as physical activity, environmental factors or socioeconomic status may still influence the observed shape differences. As such a dedicated analysis including a broader range of confounding factors would further improve our understanding of sex differences in cardiac morphology.

**Outlook**. Future work should aim to bridge sex‐specific statistical shape analysis with clinically actionable outcomes, shedding light on how sex‐specific morphological differences relate to clinical risk or therapeutic response. Applying the model to disease‐specific cohorts could help uncover sex‐specific remodelling patterns associated with heart disease (Peirlinck et al., 2021; St. Pierre et al., 2024). In addition longitudinal studies could elucidate how sex‐based anatomical differences evolve with aging or disease progression. Finally combining such large‐scale morphological analyses with physics‐based computational heart models (Moscoloni et al., 2025; Peirlinck et al., 2021, 2022; Salvador et al., 2024) accounting for functional variability (Martinez et al., 2025; Peirlinck et al., 2021) will ultimately enable a more complete understanding of sex differences in cardiac physiology and pathophysiology.

## Conclusion

Our data‐driven morphological analysis framework confirms that sex is a critical determinant of cardiac morphology, accounting for at least 25% of the variability in cardiac structure, independent of other confounders such as age, systolic blood pressure and body size. We identify Modes 1, 2 and 4 as the most discriminative shape patterns between sexes, revealing sex differences not only in overall heart size and chamber proportions but also in relative positioning and orientation of the ventricles. Importantly this study demonstrates intrinsic differences between male and female cardiac anatomy that extend beyond size and are not fully accounted for by current approaches such as allometric scaling of cardiac dimensions. Moving forward, translating our analytical framework to more diverse populations, including different ethnic backgrounds and broader age ranges, will be essential to validate and refine our findings. Further research should also focus on the implications of the observed differences in various clinical conditions and their diagnosis. Ultimately, embracing this direction will allow us to refine our understanding of cardiac morphology and enhance our ability to predict, prevent and treat heart disease in a more tailored and effective manner for both men and women.

## Additional information

## Competing interests

The authors declare they have no conflicts of interest to declare.

## Author contributions

B.M., P.S. and M.P. conceived and designed the research. J.C. and C.B. collected the data and performed the automated segmentation. B.M. conducted segmentation postprocessing, shape modelling and statistical analysis, prepared figures and drafted the manuscript. J.C., P.S. and M.P. provided feedback. P.S. and M.P. revised and edited the manuscript. B.M., C.B., J.C., P.S. and M.P. approved the final version of the manuscript. All authors have approved the final version of the manuscript and agreed to be accountable for all aspects of the work. All persons designated as authors qualify for authorship, and all those who qualify for authorship are listed.

## Funding

This work was funded by the Research Foundation – Flanders, Fonds voor Wetenschappelijk Onderzoek – Vlaanderen (Grant No. 11PS524N, to B.M.) and European Union's Horizon Europe research and innovation program (VITAL – Grant No. 101136728, to P.S. and M.P.).

## Supporting information


Peer Review History

## Data Availability

The raw imaging data and non‐imaging participant characteristics are available from UK Biobank to approved researchers via a standard application process at http://www.ukbiobank.ac.uk/register‐apply. Scripts for the shape modelling and analysis will be made available upon publication at https://github.com/peirlincklab/cardiacshapemodeling.

## Appendix A: Cutting planes calculation

To define standardised regions of interest for our shape analysis, we derive patient‐specific cutting planes based on the anatomical positions of the mitral and tricuspid valves, approximated using long‐axis atrial segmentations. The cutting process involves: (i) identifying the atrioventricular junction via directional analysis of the atrial label; (ii) constructing a cutting plane through this landmark; and (iii) retaining only the voxels below this plane in each ventricle. For each subject, we extract the long axis vector LAX, from the affine matrix of the CMR image (i.e. the matrix that maps voxel coordinates to world coordinates). Given this axis we sort the voxel positions p within the atrial label, based on their projections along LAX into a top third T and bottom third B of the atrial label sets. From the sorted voxels we computed their respective geometric centres as:

(A.1)
$$c_{\text{top}}=\frac{1}{\left|T\right|}\sum_{p\in T}p,\;c_{\text{bot}}=\frac{1}{\left|B\right|}\sum_{p\in B}p.$$

Using these centres, we define the major longitudinal axis of the atrial label m as:

(A.2)
$$m=\frac{c_{\text{top}}-c_{\text{bot}}}{∥c_{\text{top}}-c_{\text{bot}}∥}.$$

This axis is extended in both directions according to a scalar distance value d, to identify two intersection points with the atrial contour. Specifically, we use the intersection point:

(A.3)
$$\;l=c_{\text{bot}}-d\cdot m$$

to approximate the location of the atrioventricular valve. The process is carried out for both the left and right atrial labels, to identify the landmarks corresponding to the mitral and tricuspid valves, respectively. With these landmarks, we construct the cutting planes in the short‐axis view extracted from the CMR affine matrix. For each landmark $l_{j}$, we define two additional points using the SAX normal vector, s, that we use to identify the cutting planes. More specifically we use the unit vector s normal to the short axis plane, to define the offsets along two orthogonal directions, within the plane. These offsets are scaled by $d^{\prime }$ to compute the additional points:

(A.4)
$$\begin{array}{ccc} q_{1} & = & l_{j}+s\times \left[1,0,0\right]\cdot d^{\prime },\\ q_{2} & = & l_{j}+s\times \left[0,1,0\right]\cdot d^{\prime }. \end{array}$$

The points $l_{j}$, $q_{1}$ and $q_{2}$ define a cutting plane, with anchor point $l_{j}$ and cutting plane normal vector $n_{j}$:

(A.5)
$$n_{j}=\left(q_{1}-l_{j}\right)\times \left(q_{2}-l_{j}\right).$$

To perform the cutting, we calculate the world coordinates $x\in R^{3}$ of each voxel in the segmentation with respect to the original affine matrix of the segmentation. If $\left(x-l_{j}\right)\cdot n_{j}<0$ the voxel is retained; otherwise, it is discarded.

## Appendix B: Iterative closest point algorithm

Let $\Gamma_{i}$ be the i‐th surface mesh with $i\in [1,2,\cdots ,N_{P}]$, and $\Gamma_{r}$ be a reference mesh. For each surface mesh $\Gamma_{i}$, the ICP alignment algorithm seeks a rigid transformation $T_{i}=\left(R_{i},t_{i}\right)$, where $R_{i}$ is a rotation matrix and $t_{i}$ is a translation vector, which minimizes the sum of squared distances between closest points on the meshes. As such:

(A.6)
$$T_{i}=\arg\min_{R_{i},t_{i}}\sum_{k=1}^{n}{\left\|R_{i}p_{i}^{\left(k\right)}+t_{i}-p_{r}^{\left(k\right)}\right\|}^{2},$$

where $p_{i}^{\left(k\right)}$ and $p_{r}^{\left(k\right)}$ are the closest k‐th points on the meshes $\Gamma_{i}$ and $\Gamma_{r}$.

## Appendix C: Details of the LDDMM framework

Within the LDDMM framework, we handle large deformation by building diffeomorphisms Φ that use a spatially vector field $v\left(x,t\right)=v_{t}\left(x\right)$ as an instantaneous velocity field instead of a displacement field (Durrleman et al., 2014). More specifically we make our sets of control points $q\in R^{N_{q}\times 3}$ and momenta $\mu \in R^{N_{q}\times 3}$ depend on a *pseudo‐time* t over which we can integrate. Therefore the velocity field at any time $t\in \left[0,1\right]$ and space location x is written as:

(A.7)
$$v_{t}\left(x\right)=\sum_{k=1}^{N_{q}}K_{V}\left(x,q_{k}\left(t\right)\right)\mu_{k}\left(t\right),$$

where $K_{V}$ is a Gaussian kernel $K_{V}\left(x,y\right)=\exp\left({-∥x-y∥}^{2}/{\lambda_{V}}^{2}\right)$ of kernel width $\lambda_{V}$. As such the contribution of each momentum $\mu_{k}$ to the instantaneous path of the template vertex x depends on the distance between the current vertex x(t) and the control point $q_{k}\left(t\right)$ location to which the current momenta $\mu_{k}\left(t\right)$ is applied. Following this approach, any point X=x(0), located in a template geometry $\Gamma_{0}$ at pseudo‐time t=0, can be mapped $X\mapsto x\left(t\right)=\Phi_{t}\left(X\right)$, to the corresponding point x(t) in the deformed template $\Gamma_{t}$ at pseudo‐time t, by following the pseudo‐time‐varying velocity field vt's integral curve (Glaunès et al., 2008). Following Hamiltonian equations (Bône et al., 2018), we describe this velocity field by the combined time dynamics of the ensemble of control points q(t) and corresponding ensemble of momenta μ(t).

(A.8)
$$\left\{\begin{array}{c} \dot{q}\left(t\right)=K_{V}\left(q\left(t\right),q\left(t\right)\right)\cdot \mu \left(t\right)\\ \dot{\mu }\left(t\right)=-\frac{1}{2}\nabla_{q}\left\{K_{V}\left(q\left(t\right),q\left(t\right)\right)\cdot \mu {\left(t\right)}^{⊤}\mu \left(t\right)\right\} \end{array}\right.$$

For a fixed set of initial control points q(0), the time‐varying momenta μ(t) define a deformation path $\Phi_{t}$ to bring the template geometry as close to the target geometry of interest at t=1. Given that multiple paths are possible, and as such the set of time‐varying momenta μ(t) is not unique, it is natural to choose the vectors that minimise the kinetic energy along the path (Durrleman et al., 2014), namely

(A.9)
$$\frac{1}{2}\int_{0}^{1}{\left\|v_{t}\right\|}^{2}dt=\frac{1}{2}\int_{0}^{1}\mu {\left(t\right)}^{T}K_{W}\left(q\left(t\right),q\left(t\right)\right)\mu \left(t\right)dt,$$

where $K_{W}$ is a Gaussian kernel $K_{W}\left(x,y\right)=\exp\left({-∥x-y∥}^{2}/{\lambda_{W}}^{2}\right)$ of kernel width $\lambda_{W}$. The resulting paths of minimal energy, that is, the geodesic paths, are parametrised by the initial momenta $\mu_{0}=\mu \left(0\right)$. We optimise these deformation parameters $\mu_{0}$ to minimise the *relative distance*, that is, optimise the similarity, between two shape complexes. To this end we use the distance on varifolds $d_{W}$ (Charon & Trouvé, 2013) defined in the Hilbert space $W^{*}$ as:

(A.10)
$$\begin{array}{ccc} d_{W}{\left(\Gamma ,\Gamma^{\prime }\right)}^{2} & = & {\left\|\Gamma -\Gamma^{\prime }\right\|}_{W^{*}}^{2}={\left(\Gamma ,\Gamma \right)}_{W^{*}}+{\left(\Gamma^{\prime },\Gamma^{\prime }\right)}_{W^{*}}\\ & & -\;2{\left(\Gamma ,\Gamma^{\prime }\right)}_{W^{*}}, \end{array}$$

where the inner product between two meshes is defined as:

(A.11)
$${\left(\Gamma ,\Gamma^{\prime }\right)}_{W^{*}}=\sum_{p=1}^{N_{f}}\sum_{q=1}^{N_{f}^{\prime }}K_{W}\left(c_{p},c_{q}^{\prime }\right)\frac{\left(n_{p}\cdot n_{q}^{\prime }\right)}{\left|n_{p}\right|\left|{n_{q}}^{\prime }\right|}.$$

Here, $N_{f}$ and $N_{f}^{\prime }$ are the number of faces, $c_{p}$ and $c_{q}^{\prime }$ are the center of the faces and $n_{p}$ and $n_{q}^{\prime }$ are the face normals of the meshes Γ and $\Gamma^{\prime }$, respectively. Simultaneously optimising the path's kinetic energies and target shape similarities leads to the to‐be‐optimised cost function L defined in eqn (2).

## Appendix D: Metrics of the multivariate analysis of variance

The interpretation of MANCOVA results is based on the sum of squares explained by the model H and the residual sum of squares E, which represents unexplained variance. The most common statistic to evaluate the effect of predictors is derived from the eigenvalues ($\lambda_{p}$) of the matrix $A=HE^{-1}$. Specifically we use Pillai's trace statistic, which is computed as:

(A.12)
$$\Lambda_{\text{Pillai}}=\sum_{p=1,\text{…},p}\left(\frac{\lambda_{p}}{1+\lambda_{p}}\right)=\mathrm{tr}\left(A{\left(I+A\right)}^{-1}\right),$$

where p represents the number of predictors, and the eigenvalues $\lambda_{p}$ range from 0 to 1 for each predictor. Pillai's trace is calculated for each predictor individually, evaluating its effect on the dependent variables while adjusting for the other predictors. A value of 0 indicates no effect, whereas a value closer to 1 suggests that the predictor explains a substantial amount of variance in the dependent variables.

## Appendix E: Metrics of the logistic regression analysis

Table A.1 reports the pseudo‐*R* ^*2*^ and the log‐likelihood ratio (LLR) *P*‐value for all models.

**Table A.1:** Discriminatory power shape‐based logistic regression prediction of sex.

Model	pseudo‐*R* ^2^	LLR *P*‐value
Uncorrected	0.5621	5.13e‐63
BMI‐corrected	0.4830	9.08e‐53
BSA‐corrected	0.1124	1.29e‐07
Height‐corrected	0.1078	3.85e‐07
Weight‐corrected	0.1843	1.39e‐15

The pseudo‐R^2^ indicates the proportion of variability in the model explained by the model, with higher values suggesting better model fit. The LLR *P*‐value tests whether the inclusion of shape coefficients significantly improves model fit compared to a null model, with a *P*‐value less than 0.05 indicating a significant contribution of the predictors to the classification of sex.

The pseudo‐*R* ^*2*^ is calculated within the *statsmodel* package according to McFadden's pseudo‐R‐squared formulation, where the pseudo‐*R* ^*2*^ is based on the ratio between the log‐likelihood of the fitted model and that of a null model (a model with only an intercept). Specifically it reflects the proportional improvement in model fit over the null model, with higher values indicating better explanatory power (McFadden, 1974).
