# Supplementary material for: Unveiling sex dimorphism in the healthy cardiac anatomy: Fundamental differences between male and female heart shapes
Source: J Physiol. 2025 Sep 25;604(13):5512–34. doi: 10.1113/JP288667 (PMC13327772; doi:10.1113/JP288667)
Supplement: Supplementary file 1 — Peer Review History [file TJP-604-5512-s001.pdf]

The Journal of Physiology

Unveiling sex dimorphism in the healthy cardiac anatomy: fundamental differences between male and female heart shapes

Beatrice Moscoloni, Cameron Beeche, Julio A. Chirinos, Patrick Segers, and Mathias Peirlinck
DOI: 10.1113/JP288667

Corresponding author(s): Mathias Peirlinck (mplab-me@tudelft.nl)

The following individual(s) involved in review of this submission have agreed to reveal their identity: Maxx Holmes (Referee #1)

Review Timeline:

Submission Date:	27-Feb-2025
Editorial Decision:	07-May-2025
Revision Received:	28-Jun-2025
Accepted:	22-Jul-2025

Senior Editor: Bjorn Knollmann

Reviewing Editor: Viviane Timmermann

Transaction Report:

(Note: With the exception of the correction of typographical or spelling errors that could be a source of ambiguity, letters and reports are not edited. Depending on transfer agreements, referee reports obtained elsewhere may or may not be included in this compilation. Referee reports are anonymous unless the Referee chooses to sign their reports.)

Dear Dr Peirlinck,

Re: JP-RP-2025-288667 "Unveiling sex dimorphism in the healthy cardiac anatomy: fundamental differences between male and female heart shapes" by Beatrice Moscoloni, Cameron Beeche, Julio A. Chirinos, Patrick Segers, and Mathias Peirlinck

Please accept our apologies for the delay in providing you with an editorial decision.

Thank you for submitting your manuscript to The Journal of Physiology. It has been assessed by a Reviewing Editor and by 2 expert referees and we are pleased to tell you that it is potentially acceptable for publication following satisfactory major revision.

Please advise your co-authors of this decision as soon as possible.

The referee reports are copied at the end of this email.

Please address all the points raised and incorporate all requested revisions or explain in your Response to Referees why a change has not been made. We hope you will find the comments helpful and that you will be able to return your revised manuscript within 2 months. If you require longer than this, please contact journal staff: jp@physoc.org. Please note that this letter does not constitute a guarantee for acceptance of your revised manuscript.

Your revised manuscript should be submitted online using the link in your Author Tasks: Link Not Available. This link is accessible via your account as Corresponding Author; it is not available to your co-authors. If this presents a problem, please contact journal staff (jp@physoc.org). Image files from the previous version are retained on the system. Please ensure you replace or remove any files that are being revised.

If you do not wish to submit a revised version of your manuscript, you must inform our journal staff (jp@physoc.org) or reply to this email to request withdrawal. Please note that a manuscript must be formally withdrawn from the peer review process at one journal before it may be submitted to another journal.

TRANSPARENT PEER REVIEW POLICY: To improve the transparency of its peer review process, The Journal of Physiology publishes online as supporting information the peer review history of all articles accepted for publication. Readers will have access to decision letters, including Editors' comments and referee reports, for each version of the manuscript, as well as any author responses to peer review comments. Referees can decide whether or not they wish to be named on the peer review history document.

ABSTRACT FIGURES: Authors are expected to use The Journal's premium BioRender account to create/redraw their Abstract Figures. Information on how to access this account is here: https://physoc.onlinelibrary.wiley.com/journal/14697793/biorender-access.

This will enable Authors to create and download high-resolution figures. If authors have used the free BioRender service, they can use the instructions provided in the link above to download a high-resolution version suitable for publication.

The link provided should only be used for the purposes of this submission. Authors will be charged for figures created on this account if they are not related to this manuscript submission.

LANGUAGE EDITING AND SUPPORT FOR PUBLICATION: If you would like help with English language editing, or other article preparation support, Wiley Editing Services offers expert help, including English Language Editing, as well as translation, manuscript formatting, and figure formatting at www.wileyauthors.com/eeo/preparation. You can also find resources for Preparing Your Article for general guidance about writing and preparing your manuscript at www.wileyauthors.com/eeo/prepresources.

RIGOUR AND REPRODUCIBILITY: We recommend authors consult The Journal's Rigour and Reproducibility requirements to ensure that their manuscript is suitable for publication: https://physoc.onlinelibrary.wiley.com/pb-assets/hub-assets/physoc/documents/TJP-Rigour-and-Reproducibility-Requirements.pdf

REVISION CHECKLIST:

Check that your Methods section conforms to journal policy: https://jp.msubmit.net/cgi-bin/main.plex?form_type=display_requirements#methods.

Check that data presented conforms to the statistics policy: https://jp.msubmit.net/cgi-bin/main.plex?form_type=display_requirements#statistics.

Upload a full Response to Referees file. To create your 'Response to Referees': copy all the reports, including any comments from the Senior and Reviewing Editors, into a Microsoft Word, or similar, file and respond to each point, using font or background colour to distinguish comments and responses and upload as the required file type.

Please upload two versions of your manuscript text: one with all relevant changes highlighted and one clean version with no changes tracked. The manuscript file should include all tables and figure legends, but each figure/graph should be uploaded as separate, high-resolution files.

You may also upload:

- 'Potential Cover Art' for consideration as the issue's cover image
- Appropriate Supporting Information (Video, audio or data set: see https://jp.msubmit.net/cgi-bin/main.plex?form_type=display_requirements#supp).

We look forward to receiving your revised submission.

If you have any queries, please reply to this email and we will be pleased to advise.

Yours sincerely,

Bjorn Knollmann
Senior Editor
The Journal of Physiology

REQUIRED ITEMS

- Author photo and profile. First or joint first authors are asked to provide a short biography (no more than 100 words for one author or 150 words in total for joint first authors) and a portrait photograph. These should be uploaded and clearly labelled together in a Word document with the revised version of the manuscript. See Information for Authors for further details.

- The contact information for the person responsible for 'Research Governance' at your institution needs to be provided. This includes their name and an institutional email address. Please ensure the contact is not an author on this paper and provide an alternate contact if necessary, or confirm in the submission form that the author whose email was provided has sole responsibility for research governance. This is the person who is responsible for regulations, principles and standards of good practice in research carried out at the institution, for instance the ethical treatment of animals, the keeping of proper experimental records or the reporting of results.

- The reference list must be in alphabetical order, rather than numbered, to comply with our Journal format.

- Your manuscript must include a complete Additional Information section, including competing interests; funding; author contributions and acknowledgements.

- Please upload separate high-quality figure files via the submission form.

- Please ensure that any tables are editable and in Word format, and wherever possible, embedded in the article file itself.

- Please ensure that the Article File you upload is a Word file.

- A Data Availability Statement is required for all papers reporting original data. This must be in the Additional Information section of the manuscript itself. It must have the paragraph heading 'Data Availability Statement'. All data supporting the results in the paper must be either: in the paper itself; uploaded as Supporting Information for Online Publication; or archived in an appropriate public repository. The statement needs to describe the availability or the absence of shared data. Authors must include in their statement: a link to the repository they have used, or a statement that it is available as Supporting Information; reference the data in the appropriate sections(s) of their manuscript; and cite the data they have shared in the

References section. Whenever possible, the scripts and other artefacts used to generate the analyses presented in the paper should also be publicly archived. If sharing data compromises ethical standards or legal requirements then authors are not expected to share it, but must note this in their statement. For more information, see our Statistics Policy.

- Please include an Abstract Figure file, as well as the Figure Legend text within the main article file. The Abstract Figure is a piece of artwork designed to give readers an immediate understanding of the research and should summarise the main conclusions. If possible, the image should be easily 'readable' from left to right or top to bottom. It should show the physiological relevance of the manuscript so readers can assess the importance and content of its findings. Abstract Figures should not merely recapitulate other figures in the manuscript. Please try to keep the diagram as simple as possible and without superfluous information that may distract from the main conclusion(s). Abstract Figures must be provided by authors no later than the revised manuscript stage and should be uploaded as a separate file during online submission labelled as File Type 'Abstract Figure'. Please also ensure that you include the figure legend in the main article file. All Abstract Figures should be created using BioRender. Authors should use The Journal's premium BioRender account to export high-resolution images. Details on how to use and access the premium account are included as part of this email.

EDITOR COMMENTS

Reviewing Editor:

Whilst generally very positive, the reviewers identified a number of clarity and modelling issues that need to be addressed, including: improving the interpretation of results in clinical and physiological context; expanding and improving the discussion section; addressing study aims more fully, particularly the effects of age, body size and blood pressure; improving the integration and discussion of relevant literature; improving figures, tables and methodological clarity; addressing confounders; and expanding limitations.

Please also see 'Required Items' above.

Senior Editor:

Both reviewers and reviewing editor found merit in the work, but identified several deficiencies that would need to be addressed before the manuscript can be considered for publication.

REFEREE COMMENTS

Referee #1:

This study presents a strong, data-driven framework for analysing the morphological variation in cardiac anatomies. Utilising high-power approaches, the authors generate a template cardiac anatomy and assess the minimum transformations necessary to transform this template to each of 456 cardiac meshes produced from CMR imaging data in the UK Biobank. The authors use this framework to produce several statistical shape models, correcting for age, systolic blood pressure, body size, BMI, etc. to assess the role of sex as a critical determinant of cardiac morphology. Findings reveal that sex alone explains at least 25% of morphological variability, potentially more, with strong discrimination between males and females across cardiac chamber volumes, anterior-posterior width of the RV and relative positioning of cardiac chambers. These differences persist when correcting for age, BMI, height, weight and systolic BP.

General Comments:

This paper addresses a very important research question, the models are generated in a rigorous manner, and the data they derive have the potential to be utilised for real clinical impact.

However, the introduction falls short of explaining the current utilisation of the clinical measurements described in the text. Additional details as to how echocardiographic or electrocardiogram biomarkers are included in, or affected by, shape and size characteristics of the heart and/or patient aren't sufficiently described, nor is there relevancy in cardiac pathologies. Some examples would be Shiwani et al. (2025) HCM guidelines, which discusses sex differences in maximal wall thickness, or the modelling and simulation studies which link ventricular size with ECG such as Mincholé et al. 2019. Details of previous works in this area would be beneficial to the manuscript, previous studies of sex differences in cardiac geometry, a description of biological mechanisms (i.e. Koenig et al. (1982) links cardiac size differences with testosterone in mice). Lastly, the aim "to assess the impact of age, body size, BP on morphology" is great, but was not adequately evidenced in

the rest of the manuscript.

The manuscript is highly technical, and the results of the machine learning and technical analysis are very well explained and clear. Unfortunately, the manuscript falls short of interpreting the results of these machine learning and statistical approaches to a satisfactory level. Examples of this are in Figures 7 & 8, Tables 2 & 3. Descriptions on how the male and female anatomies are significantly different, what that means for the shape of the heart, translations to physiological measurements in the mesh (e.g. length vs width ratio) to show not just statistical significance, but clinical significance would elevate this work significantly. There are terms throughout the paper, such as "differences in the relative positioning of the ventricles" which are not adequately described as to what these differences in positioning are, or what it means clinically.

Similarly, while the methods are exceedingly clear, the manuscript fails to explain the relevance of the use of such techniques. One key example of this, is the second step of the cost-minimisation algorithm presented in Section 2.2.1; while the background knowledge required to understand this method is precise and clear - there is no mention of why this is required in the context of developing the statistical shape models or its physiological relevance.

The comparison with Burns et al. (2024) in the discussion presents an issue with your interpretation of mode 3; it could be that the use of this mode of a metric of longitudinal length is different, but this could be directly measured on your meshes instead of relying on black box machine learning. This difference could also be because Burns et al. (2024) presents an analysis with 100-fold more participants. Due to the relevancy of this study to your paper, I believe it should have been included in the introduction of the paper to further motivate the need for a study of this kind, without confounding factors introduced through pathologies.

Additionally, the limitations section needs to be heavily expanded. Reading it, it seemed like the authors glossed over some limitations mentioned elsewhere, such as the lack of ethnicity included in this study which is mentioned in the conclusions. The UKBB cohort for example has differences even compared to the native UK population, e.g. leaner and taller (Schoeler et al. 2023). The further directions presented also need to be more interesting. Describe how you could do more studies that would have clinical influence, rather than how to improve the current study. Those should be discussed as a limitation instead.

This study has great potential, it's excellent and extremely thorough work - but it requires further analysis of the results and interpretation to give the manuscript more clinical and physiological significance. The discussion can be improved significantly.

Specific comments:

• Figure 1 needs to have larger panels, it's very difficult to see even zoomed to full screen.

• Bai et al. needs to be mentioned by name in 2.1.2 to make it easily understandable that this work comes from that paper.

• Table 1 should include ethnicity.

• Figure 2 is really good, very clear and informative.

• In 2.2, statements of the intended outcome would benefit the description of the methodology.

• In 2.4; did you check for equal variance with Hotelling's test? Similarly, did you check the normality of the principle components for MANCOVA?

• Figure 4 seems unnecessary without further information of why this method was performed.

• Figure 5 is good, very clear and justifies your decision-making.

• Figure 6 is good, but needs to have larger panels. It's difficult to see the differences beyond mode 3, and more so after mode 6. Perhaps fewer modes, or two rows of modes with larger panels would improve the visibility?

• Figure 7 and the text in 3.2. Firstly, the data itself in Figure 7 is presented very nicely and the violin plots are clear, but several improvements are suggested:

o Description is needed pertaining to how these modes are different and what this translates to in terms of cardiac morphology. Translation to quantitative measurements (or differences between sexes) in the mesh, e.g. length vs width ratio.

o Annotations on the figure to highlight statistically significant differences in the mean, e.g. an arrow to the larger mean, star values for significance, etc.

o Make black bars and mean points visible, or remove them.

• Figure 8

o There is a strong need for further explanation as to the significance of the information presented in this figure. As it stands, it doesn't really give much translatable insights.

o It requires a legend. It's not sufficient for the reader to need to go back and forth between the figure and text to match the (non-matching in significance) quantification of anatomical variance with the text in the figure to identify which is which.

o It would also be interesting to see if the progression of heart shape with age was sex dependent here.

• Tables 2&3

o Unsure of what these tables mean; from my understanding, it shows men are significantly different from women which would be the case as I believe this includes cardiac size?

o I'm not sure these are necessary. Perhaps as an appendix to justify the choice of "the best model" for sex-discrimination.

• Figure 10 and Table 4

o Difficult to visualise.

o Are all five models necessary here? It detracts from the information provided in the figure. I think instead perhaps justify which model is better and then only visualise this model.

o Mode 7 in Figure 10 shows 3 anatomies which are in the same perspective orientation as Mode 6 for the uncorrected, BSA and Height corrected models. Is this explainable, or an error?

o Panels need to be bigger, hard to see the differences in the purple shading panels.

• In the discussion, there is lots of repetition from the methods and results.

• There is a lack of actionable consequences or clinical evaluation in the discussion. Some considerations of how the differences presented in the interpretation of the key modes would manifest in clinically relevant scenarios (treatment, pathology, diagnosis, biomarkers) would improve the discussion greatly.

• The comparison with Burns et al. (2024) regarding the largest principle components of each study is interesting, and I would like to see the authors expand further insights into these differences; and where the other sex-significant modes observed in this study compare with Burns et al. (2024).

Referee #2:

Sex has been increasingly recognized as an independent biological variable influencing cardiovascular disease risk. While the link between sex and cardiac anatomical features has been established, studies quantitatively assessing its impact independent of common confounders-such as age and body metrics-remain scarce. In this study, Drs. Moscoloni and colleagues conducted a computational analysis using statistical shape modeling to investigate sex-based differences in healthy cardiac anatomy. Their results demonstrate that sex explains at least 25% of anatomical variability, primarily affecting cardiac chamber volume, the anterior-posterior width of the right ventricle, and the relative positioning of the chambers. These findings provide valuable insights into the role of sex in cardiac structure.

Overall, the study's methodology is well justified, and the manuscript presents a reasonable amount of data supporting its conclusions. However, some limitations should be noted: 1) Narrow confounder adjustment - The analysis did not account for other potential confounders beyond age and body size. 2) Methodological clarity - Some descriptions of the analytical approach are unclear. 3) Readability - The highly quantitative nature of the study makes the manuscript challenging to follow in places. These weaknesses are explained as follows.

Major points:

• The analysis examines associations between biventricular shape statistics and factors such as sex, age, blood pressure, and body metrics. However, the inclusion of covariates appears insufficient. Notably, lifestyle factors (e.g., smoking, alcohol use) - known risk modifiers for cardiac pathology - were not incorporated. Clarifying whether such variables influence shape statistics would strengthen the interpretation of sex-specific effects and improve clinical relevance.

• While MANCOVA was appropriately chosen to assess shape coefficient associations, its validity depends on the normality of dependent variables. The manuscript does not explicitly state whether normality testing was performed or how potential violations were addressed. Documentation of these methodological checks would enhance the robustness of the statistical approach.

• The manuscript in its current form appears hard to understand. This is partially because the study is very quantitative with several different analytical approaches that are not routinely performed in physiology. For example, given that the statistical shape analysis is the core analytical approach in this study, some intermediate steps might be helpful considering the broad readership of this journal. Also, in multiple occasions descriptions of the approach appear unclear. Some examples include:

- Page 4, Step 2: Motion correction is mentioned but not clearly described. In addition, it is unclear whether the β-VAE approach implements motion correction in addition to up-sampling. Some descriptions in Methods appears unclear and could benefit from figure illustrations. For instance, while a region of interest was defined to standardize the subsequent analysis, only landmark descriptions are provided for Step 3.

- Some approaches may warrant more detailed descriptions of their justifications. For example, the minimization of the cost function in Eq. 2 was achieved through a two-step iteration (page 6). However, it remains unclear whether these steps were adapted from previous studies without modifications or improved by the authors, and why these particular approaches were chosen.

- For the LDDMM approach described on page 6: Are the results of the anatomical mapping and reconstruction and the subsequent analysis dependent on the original input "template"? While Eq. 5 describes an average template, it is unclear how this template was obtained. Was it generated through the two-step optimization described under Eq. 2? More consistent descriptions would help the reviewer better follow the methodology. Additionally, note that "Γ_0" represents the 'derived' template in Fig. 4. The manuscript would benefit from more consistent and distinguishable notation for "template" at different processing steps.

- The statement: "Of the top 10 hyperparameter combinations evaluated, we qualitatively based our choice on the trade-off between low reconstruction error, avoiding too local and unrealistic deformations, and limiting run time, arriving at λ_V = 10 mm and λ_W = 6 mm" could be strengthened with examples or illustrations to help readers understand why/how these specific hyperparameters were optimal.

• The authors are encouraged to have a data/code availability statement/sharing plan should this manuscript be revised.

Minor

• Eq. 3, 1/N should be 1/N_v instead?

• Eqs. 5 and 6 both describe \alpha_i, however they seem to represent distinct variables. Thus it would be helpful to distinguish the two by using different subscripts.

• In Fig 4, how different quantitatively are those two templates?

• Fig 8, the 'four' different models should be labelled explicitly.

• Please describe how pseudo-R^2 was computed in the logistic regression analysis, given that multiple ways of pseudo-R2 have been documented (https://stats.oarc.ucla.edu/other/mult-pkg/faq/general/faq-what-are-pseudo-r-squareds/).

END OF COMMENTS

Response to the Reviews

Unveiling sex dimorphism in the healthy cardiac anatomy: fundamental differences between male and female heart shapes

Manuscript Reference Number:
JP-RP-2025-288667

Authors:
Beatrice Moscoloni
Cameron Beeche
Julio A. Chirinos
Patrick Segers
Mathias Peirlinck

Date: June 27th, 2025

We would like to thank the senior editor, the reviewing editor and both reviewers for the constructive comments and feedback, which have allowed us to improve the quality of the manuscript. We have addressed the questions and incorporated the suggestions in the revised manuscript. All edits are colored in red in the revised manuscript to differentiate with contents in the original manuscript.

Response To Editors

Reviewing Editor

> Whilst generally very positive, the reviewers identified a number of clarity and modelling issues that need to be addressed, including: improving the interpretation of results in clinical and physiological context; expanding and improving the discussion section; addressing study aims more fully, particularly the effects of age, body size and blood pressure; improving the integration and discussion of relevant literature; improving figures, tables and methodological clarity; addressing confounders; and expanding limitations.

Response

We would like to thank the reviewing editor for collecting and relaying the review responses to us. We are resubmitting the revised manuscript with the aim of overcoming the issues that were pointed out from the reviewers. More specifically:

- We elaborated on the presented results in more details and provided further interpretation with respect to the physiological meaning of the observed results.
- We have expanded our discussion with respect to the possible clinical implications of the observed results.
- We have included additional literature in both our introduction and our discussion.
- We have rephrased our study objective, to maintain a clear focus on the dedicated analysis of sex differences (i.e., after correcting for other confounders, such as blood pressure, age, and body size) and to clarify the intended focus of the study.
- We have improved our figures and tables, as well as clarified the intended outcome of several steps of our methodology.
- We have extended both the limitations and the outlook sections.

We hope that the revisions are satisfactory and that the manuscript can be accepted for publication in its updated form.

Senior Editor

> Both reviewers and reviewing editor found merit in the work, but identified several deficiencies that would need to be addressed before the manuscript can be considered for publication.

Response

We would like to thank the senior editor for assessing our work. We have carefully addressed the concerns raised by both reviewers and the reviewing editor, and we hope that the updated version of the manuscript satisfactorily resolves the identified deficiencies.

Response To Reviewer #1

Overall Comments

> This study presents a strong, data-driven framework for analysing the morphological variation in cardiac anatomies. Utilising high-power approaches, the authors generate a template cardiac anatomy and assess the minimum transformations necessary to transform this template to each of 456 cardiac meshes produced from CMR imaging data in the UK Biobank. The authors use this framework to produce several statistical shape models, correcting for age, systolic blood pressure, body size, BMI, etc. to assess the role of sex as a critical determinant of cardiac morphology. Findings reveal that sex alone explains at least 25% of morphological variability, potentially more, with strong discrimination between males and females across cardiac chamber volumes, anterior-posterior width of the RV and relative positioning of cardiac chambers. These differences persist when correcting for age, BMI, height, weight and systolic BP.

Response

We appreciate the careful review and positive feedback on the value of our contribution, as well as the nice summary of the key points of our analysis. We highly appreciate your review and have carefully addressed your questions and remarks on the presented work. We hope that you find the following response and the revised sections satisfactory.

Reviewer Comment R1.1.

> This paper addresses a very important research question, the models are generated in a rigorous manner, and the data they derive have the potential to be utilised for real clinical impact.
>
> However, the introduction falls short of explaining the current utilisation of the clinical measurements described in the text. Additional details as to how echocardiographic or electrocardiogram biomarkers are included in, or affected by, shape and size characteristics of the heart and/or patient aren't sufficiently described, nor is there relevancy in cardiac pathologies. Some examples would be Shiwani et al. (2025) HCM guidelines, which discusses sex differences in maximal wall thickness, or the modelling and simulation studies which link ventricular size with ECG such as Mincholé et al. 2019. Details of previous works in this area would be beneficial to the manuscript, previous studies of sex differences in cardiac geometry, a description of biological mechanisms (i.e. Koenig et al. (1982) links cardiac size differences with testosterone in mice). Lastly, the aim "to assess the impact of age, body size, BP on morphology" is great, but was not adequately evidenced in the rest of the manuscript.

Response

We thank the reviewer for the insightful comment and for the relevant literature they pointed us to. We acknowledge that the current version of the manuscript was falling short in the description of how current cardiac biomarkers are affected by variability in shape and size between patients. As such, we have extended our introduction as follows:

Moreover, shape and anatomical assessments in these studies, and more generally in clinical practice, often rely on simple two-dimensional morphological parameters, such as lengths and diameters (Westaby et al. 2023). Such approaches overlook the rich data provided by current imaging techniques and underestimate the intrinsic complexity of global and local shape features in cardiac anatomy, and its impact on clinical biomarkers (Bruse et al. 2016; Mincholé et al. 2019; Shiwani et al. 2025). For instance, a recent population-based study challenged the *one-size-fits-all* threshold of ≥15 mm for maximum wall thickness in hypertrophic cardiomyopathy diagnosis, showing a substantial sex skew in clinical classification (Shiwani et al. 2025). Similarly, ventricular geometry has been shown to significantly affect electrocardiography (ECG) biomarkers, underscoring the need to account for anatomical variation in ECG-based diagnostic models (Mincholé et al. 2019). Consequently, a comprehensive morphological analysis that accounts for potential confounding variables is crucial for advancing our understanding of sex differences in cardiac morphology, and their implications for diagnosis and treatment of cardiovascular disease (Gao et al. 2019).

With respect to the study aim, we agree that the phrasing of the second study aim in the present form is not fully in line with the results that are presented in the manuscript. Given that our main goal for this work was to focus on assessing sex differences in cardiac morphology, while accounting for the effect of confounders, we rephrased the study aims as follows:

- Examine how morphological cardiac anatomy descriptors differ between the male and female subgroups.
- Quantify the impact of sex on cardiac morphology, after accounting for age, body size, and blood pressure.
- Identify the cardiac shape components that best distinguish male and female populations, after accounting for confounders.

Reviewer Comment R1.2.

> The manuscript is highly technical, and the results of the machine learning and technical analysis are very well explained and clear. Unfortunately, the manuscript falls short of interpreting the results of these machine learning and statistical approaches to a satisfactory level. Examples of this are in Figures 7 & 8, Tables 2 & 3. Descriptions on how the male and female anatomies are significantly different, what that means for the shape of the heart, translations to physiological measurements in the mesh (e.g. length vs width ratio) to show not just statistical significance, but clinical significance would elevate this work significantly. There are terms throughout the paper, such as ”differences in the relative positioning of the ventricles” which are not adequately described as to what these differences in

positioning are, or what it means clinically.

Response

We thank the reviewer for the very relevant comment and suggestions with respect to the clinical interpretation of the results of our analysis. We are fully aware and agree that direct translation of the presented modes of variation to physiological measurements would further elevate our result section. We would like to clarify the underlying reasoning to the current interpretation of our results; on the matter of clinical interpretation of the modes, we stay on the conservative side in our clinical interpretation for two main reasons:

1. While principal component analysis (i.e., in statistical shape modeling applications) in principle aims to provide modes of anatomical variation that are as disentangled as possible, in many cases this disentanglement does not directly translate into fully disentangled physiologically measurable metrics. As such, one mode might be mostly related to a specific measurement (e.g. length-width ratio), while still retaining small contributions from other measurements. Therefore, linking directly the modes to individual clinical measurements would partially oversimplify the morphological complexity that these modes carry.
2. While we can currently visually associate the modes with certain morphological feature (size, elongation, etc.) by means of visualization of the shape changes along multiple standard deviation (i.e., Section 3.1), we believe that only quantitative correlation between the shape coefficients and validated measured metrics on each subject mesh would allow to provide a strong foundation for clinical translation of our findings.

In response to the reviewer's suggestion, we thoroughly expanded our results (sub)sections as follows:

Section 3.2 - We elaborated on the implications of the statistical observations on the violin plots, by linking them to the visual interpretation from Figure 6:

The violin plots in Figure **??** show the distribution of the first eight shape coefficients, stratified by sex. Upon visual inspection, we observe that modes 1 and 2 show significant differences in the mean shape distributions between males and females. Considering the visual interpretation of these modes from Figure 7, these differences suggest significant sex-specific variation in mean overall chamber size (mode 1), as well as the transverse width of the right ventricle and septum (mode 2). Modes 4 also shows a statistically significant difference, suggesting sex-specific differences in the relative orientation of the RV respective to the LV, although less pronounced than the first two modes. While mode 5 also exhibit noticeable visual differences in both means and variances, the difference is not statistically significant, altough it might reflect heterogeneity in RV obliqueness, particularly among males. Modes 6 and 7 again reveal subtler variations, particularly in the tails of their distributions and mean values, suggesting that males present more extreme anatomical variations in terms of LV bulging (mode 6) and RV sphericity (mode 7). However, these differences do not appear to be statistically significant based on the independent t-test result.

Section 3.3 - We clarified the practical implication of Pillai's trace values from MANCOVA analysis, with respect to anatomical variance explained by sex:

From the MANCOVA conducted on the first 20 shape coefficients, we observe that all explanatory variables, including BMI, BSA, height, weight, age, and systolic blood pressure, significantly affect overall cardiac morphology, with $p \ll 0.001$ across all variables and models. However, their

contributions to explaining cardiac morphological variance, quantified by Pillai's trace (Figure 8), are generally smaller than that of sex. Specifically, the variance in the shape coefficients explained by sex, after accounting for other confounders, ranges from 0.25 in the height-model to 0.55 in the BMI-model, demonstrating that sex consistently accounts for a substantial portion of cardiac morphological variability.

Section 3.4 - We clarified the practical implication of the results of Hotelling's T2 test, with respect to overall sex differences in cardiac morphology:

The T^2 statistics reveal that corrections using age, systolic blood pressure, and height (height-corrected model) result in the smallest differences in the mean shape coefficients between the two groups, whereas the BMI-model leads to differences closer to the uncorrected model. Additionally, BSA-corrected shape coefficients follow a trend similar to that of the height-corrected coefficients, while a combined age, systolic blood pressure and weight correction (weight-corrected model) yields less pronounced differences in the means compared to BSA- and height-corrected shapes, yet still considerable lower than those seen in the uncorrected and BMI-corrected models. These results imply that statistically significant sex-differences are present in the multivariate collection of morphological descriptions, regardless of the correction for confounders.

With respect to the interpretation of the modes of variation, we provide Supplementary Material with animated visualizations that further elucidate the provided interpretation present in Section 3.1. Additionally, to further elucidate the interpretation of mode 4, and coherently with other published studies in literature (Burns et al. 2024), we have rephrased "relative positioning of the ventricles" to "relative orientation of the RV respective to the LV".

Reviewer Comment R1.3

> Similarly, while the methods are exceedingly clear, the manuscript fails to explain the relevance of the use of such techniques. One key example of this, is the second step of the cost-minimisation algorithm presented in Section 2.2.1; while the background knowledge required to understand this method is precise and clear - there is no mention of why this is required in the context of developing the statistical shape models or its physiological relevance.

Response

We thank the reviewer for the very relevant comment on the necessity and role of optimizing the template geometry within our framework.

This second step — optimization with respect to the (initial) template geometry $\mathbf{\Gamma}$ - is needed for two main reasons:

1. From the technical standpoint, iteratively optimizing the template improves the estimation of the subject-specific momenta applied to the template at each iteration. As the template converges toward the mean shape of the population, it becomes easier for the subject-specific momenta to deform it to match the individual target geometries more accurately (see Figure 3).

2. From the physiological standpoint, updating the template ensures that the final template geometry represents a population-wide anatomical baseline. This optimized template then acts as a stable, central reference point in the shape space, from which individual anatomical variations can be meaningfully quantified and analyzed using PCA.

We have clarified the practical purpose of iterating both optimization steps in our manuscript. Our revised Section 2.2.1 reads as follows:

$$\mathcal{L}\left(\boldsymbol{\Gamma}, \boldsymbol{\mu}\right) = \sum_{i=1}^{N_{\mathcal{P}}} \frac{1}{\sigma^2} d_W \left(\boldsymbol{\Phi}_{\mathbf{q},\boldsymbol{\mu}_i}\left(\boldsymbol{\Gamma}\right), \boldsymbol{\Gamma}_i\right)^2 + \sum_{i=1}^{N_{\mathcal{P}}} \boldsymbol{\mu}_i^{\top} K_W(\mathbf{q}, \mathbf{q}) \boldsymbol{\mu}_i, \tag{1}$$

To optimize Eq. 1, we iterate until convergence (Bône et al. 2018): (i) Optimization with respect to the momenta $\boldsymbol{\mu}$ while keeping the template $\boldsymbol{\Gamma}$ fixed, yielding the $N_{\mathcal{P}}$ deformations that map the (current) template to each target geometry. (ii) Optimization with respect to the template $\boldsymbol{\Gamma}$ while keeping the momenta $\boldsymbol{\mu}$ fixed, resulting in an updated template derived from the (current) set of subject-specific momenta. The first step computes the subject-specific deformations needed to match the template to each target anatomy, establishing point-to-point correspondence between the template and the target mesh. The second step ensures that the final template reflects a population-wide anatomical baseline, facilitating both physiological interpretability and more accurate shape modeling. For further details about the LDDMM framework and the optimization approach we refer to the original work (Durrleman et al. 2014; Bône et al. 2018). Once optimized, the cohort of geometries is represented by the final template $\boldsymbol{\Gamma}$ and a unique set of momenta $\boldsymbol{\mu}_i$ for each subject, i.e., target geometry i. Applying these momenta to the template vertices reconstructs the corresponding biventricular surface meshes for each subject, ensuring consistent point-to-point correspondence across all meshes in the cohort.

Reviewer Comment R1.4

> The comparison with Burns et al. (2024) in the discussion presents an issue with your interpretation of mode 3; it could be that the use of this mode of a metric of longitudinal length is different, but this could be directly measured on your meshes instead of relying on black box machine learning. This difference could also be because Burns et al. (2024) presents an analysis with 100-fold more participants. Due to the relevancy of this study to your paper, I believe it should have been included in the introduction of the paper to further motivate the need for a study of this kind, without confounding factors introduced through pathologies.

Response

We thank the reviewer for this valuable observation regarding the interpretation of mode 3 and the comparison to the findings by Burns et al. (2024). Both our study and that of Burns et al. rely on PCA-based shape modeling of biventricular geometries, with the interpretation of the modes relying solely on visual description. As such, the definition and extraction of shape modes follow similar methodologies. That said, we agree that while in both cases the modes were visually interpreted as reflecting longitudinal ventricular elongation, this might still include some uncertainty in the interpretation due to the "black-box" nature of PCA, and the inherently subjective visual interpretation. Concomitantly, we acknowledge that the sample size discrepancy is considerable:

Burns et al. analyze over 45,000 subjects, while our study uses a focused, and thus smaller health-screened cohort. It is therefore possible that differences in statistical power may account for the divergent findings with respect to mode 3.

We have further underscored this in our discussion as follows:

While our visual interpretation of mode 3 captures variations in ventricular longitudinal length, we do not observe a significant contribution of this mode to sex-based differentiation. Notably, the aforementioned analysis included more than 45,000 subjects from the UK Biobank population without health-based selection criteria. As a result, the observed sex-specific trend in ventricular elongation may either reflect disease-related remodeling rather than inherent differences in healthy cardiac anatomy, or result from differing sample sizes between the studies.

Additionally, we appreciate the reviewers suggestion and have added a reference to Burns et al. in our introduction as follows:
In turn, this atlas enables a more complex and comprehensive data-driven analysis of morphological variation than is possible with traditional measurements (Govil et al. 2023; Suinesiaputra et al. 2018). For example, a recent large-scale study constructed a heart shape atlas from UK Biobank participants to identify genetic determinants of cardiac shape and associate specific shape components to increased cardiometabolic disease risk (Burns et al. 2024). Nevertheless, a dedicated statistical shape analysis explicitly addressing sex differences in cardiac morphology within a healthy population is still lacking.

In this work, we apply a data-driven morphological analysis to investigate sex differences in cardiac anatomy within a healthy subset of the UK Biobank imaging population (Littlejohns et al. 2020).

Reviewer Comment R1.5

> Additionally, the limitations section needs to be heavily expanded. Reading it, it seemed like the authors glossed over some limitations mentioned elsewhere, such as the lack of ethnicity included in this study which is mentioned in the conclusions. The UKBB cohort for example has differences even compared to the native UK population, e.g. leaner and taller (Schoeler et al. 2023). The further directions presented also need to be more interesting. Describe how you could do more studies that would have clinical influence, rather than how to improve the current study. Those should be discussed as a limitation instead.

Response

We thank the reviewer and agree that the limitations section as it stand needs to be expanded. We have extended it to include relevant limitations related to UKBB cohort as well as our selected subcohort.

The revised limitation section reads as follows:

Limitations. While this study presents a novel statistical shape analysis to investigate sex differences in cardiac anatomy, several limitations should be acknowledged. First, we selected a reasonably large cohort of healthy subjects from the UK Biobank population, enabling us to focus on baseline anatomical sex differences. However, our analysis was restricted to individuals of Caucasian ethnicity, which may limit the generalizability of our results to other ethnic groups. Extending the

analysis to a larger and more diverse population that includes different ethnic backgrounds, would enhance the applicability of our findings. Second, although we corrected for key demographic and physiological confounders (e.g., age, body size, systolic blood pressure), unmeasured variables such as physical activity, environmental factors, or socioeconomic status may still influence the observed shape differences. As such, a dedicated analysis including a broader range of confounding factors would further improve our understanding of sex differences in cardiac morphology.

Additionally, we have rephrased and expanded a dedicated outlook section to provide future directions:

Outlook. Future work should aim to bridge sex-specific statistical shape analysis with clinically actionable outcomes, shedding light on how sex-specific morphological differences relate to clinical risk or therapeutic response. Applying the model to disease-specific cohorts could help uncover sex-specific remodeling patterns associated with heart disease (Peirlinck et al. 2021; St. Pierre et al. 2024). Additionally, longitudinal studies could elucidate how sex-based anatomical differences evolve with aging or disease progression. Finally, combining such large-scale morphological analyses with physics-based computational heart models (Peirlinck et al. 2021; Peirlinck et al. 2022; Salvador et al. 2024) accounting for functional variability (Peirlinck, Costabal, and Kuhl 2021; Martinez et al. 2025) will ultimately enable a more complete understanding of sex differences in cardiac physiology and pathophysiology.

Reviewer Comment R1.6

> This study has great potential, it's excellent and extremely thorough work - but it requires further analysis of the results and interpretation to give the manuscript more clinical and physiological significance. The discussion can be improved significantly.

Response

We thank the reviewer for the very supportive comment. We believe their thorough and relevant comments have greatly improved the manuscript, especially in the introduction, results, and discussion sections. We further improved the discussion with more clinically and physiologically relevant insights:

This result indicates a clear separation between the two multivariate distributions, reinforcing the presence of substantial sex differences in cardiac anatomy. Additionally, as shown in Figure 7, differences are present in both the means, variance and tails of the derived morphological descriptors. This evidence suggests that sex differences in cardiac anatomy are not limited to average shape but span the entire morphological distribution. This implies that female and male hearts not only differ in typical geometry but also in the range and variability of possible anatomical configurations, further highlighting the need for sex-specific reference models in both clinical and research setting (Kruk 2025).

While the variability attributed to body size varies based on the specific body size metric considered (i.e., BMI, BSA, height or weight), sex consistently accounts for at least 25% of the variability –

even after accounting for age, systolic blood pressure and body size. This finding aligns with a recent ex-vivo morphological analysis on normal hearts, which analyzed the dimensions of several cardiac structures, while accounting for sex, age, and body measurements (Westaby et al. 2023). The study identified sex as the strongest predictor of cardiac anatomy. Our results reinforce this conclusion through a data-driven statistical shape analysis, conducted on in-vivo imaging data. Unlike conventional measurements as diameter, lengths and volumes, our approach captures complex three-dimensional variations, offering a more nuance and comprehensive characterization of sex-based differences in cardiac anatomy. Furthermore, recognizing the magnitude of this sex effect supports the need for sex-stratified analyses, and the need to include sex-based anatomical variability in diagnostic pathways.

In a broader sense, the persistence of cardiac morphology discrimination underscores the presence of fundamental anatomical differences that extend beyond artifacts of confounding variables. These differences are likely rooted in genetic or developmental factors inherent to each sex, rather than being mere consequences of body size disparities (Martin and Leinwand 2024; Winham, Andrade, and Miller 2015; Lin et al. 2023; Burns et al. 2024; Reue and Wiese 2022). Nonetheless, accounting for these intrinsic differences in clinical biomarkers and imaging-based diagnostic thresholds (Shiwani et al. 2025) will be essential to reduce sex-related disparities in cardiovascular care.

Reviewer Comment R1.7

Specific comments:

1. Figure 1 needs to have larger panels, it's very difficult to see even zoomed to full screen.
2. Bai et al. needs to be mentioned by name in 2.1.2 to make it easily understandable that this work comes from that paper.
3. Table 1 should include ethnicity.
4. Figure 2 is really good, very clear and informative.
5. Statements of the intended outcome would benefit the description of the methodology.
6. Did you check for equal variance with Hotelling's test? Similarly, did you check the normality of the principle components for MANCOVA?
7. Figure 4 seems unnecessary without further information of why this method was performed.
8. Figure 5 is good, very clear and justifies your decision-making
9. Figure 6 is good, but needs to have larger panels. It's difficult to see the differences beyond mode 3, and more so after mode 6. Perhaps fewer modes, or two rows of modes with larger panels would improve the visibility?
10. Figure 7 and the text in 3.2. Firstly, the data itself in Figure 7 is presented very nicely and the violin plots are clear, but several improvements are suggested:

- Description is needed pertaining to how these modes are different and what this translates to in terms of cardiac morphology. Translation to quantitative measurements (or differences between sexes) in the mesh, e.g. length vs width ratio.
- Annotations on the figure to highlight statistically significant differences in the mean, e.g. an arrow to the larger mean, star values for significance, etc.
- Make black bars and mean points visible or remove them.

11. Figure 8:
    - There is a strong need for further explanation as to the significance of the information presented in this figure. As it stands, it doesn't really give much translatable insights.
    - It requires a legend. It's not sufficient for the reader to need to go back and forth between the figure and text to match the (non-matching in significance) quantification of anatomical variance with the text in the figure to identify which is which.
    - It would also be interesting to see if the progression of heart shape with age was sex dependent here.
12. Tables 2&3:
    - Unsure of what these tables mean; from my understanding, it shows men are significantly different from women which would be the case as I believe this includes cardiac size?
    - I'm not sure these are necessary. Perhaps as an appendix to justify the choice of "the best model" for sex-discrimination.
13. Figure 10 and table 4:
    - Difficult to visualise.
    - Are all five models necessary here? It detracts from the information provided in the figure. I think instead perhaps justify which model is better and then only visualise this model.
    - Mode 7 in Figure 10 shows 3 anatomies which are in the same perspective orientation as Mode 6 for the uncorrected, BSA and Height corrected models. Is this explainable, or an error?
14. Panels need to be bigger hard to see the differences in the purple shading panels.

Response

We thank the reviewer for the very detailed review and for the very useful comments and suggestions, which we have addressed point by point:

1. Figure 1: We have modified the figure with increased size and increased fonts to improve the visibility of the panels.

2. We have modified the relevant part of the manuscript to underline that we use an available, pre-trained neural network, as follows:

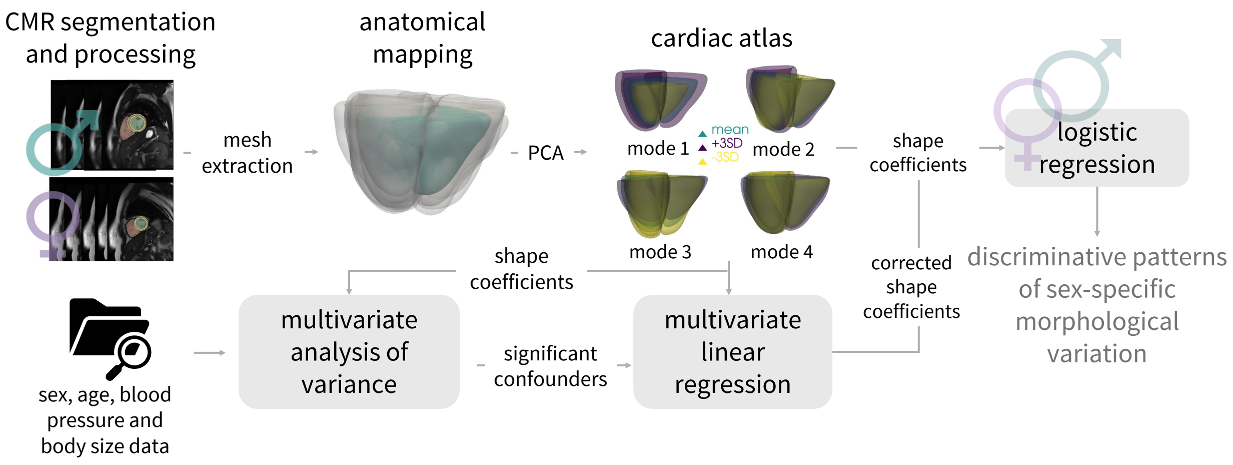

We apply a pre-trained, openly-available fully convolutional neural network (Bai et al. 2018) to segment five cardiac structures of interest: the left ventricular (LV) and right ventricular (RV) blood pools, the LV myocardium, and left and right atrial blood pools.

Additionally, we have changed the in-text citation style to APA (i.e., Author, year), in line with the Journal of Physiology style.

3. We appreciate the reviewer's suggestion on including ethnicity in Table 1. However, we chose not to report ethnicity in Table 1 in order to maintain the structure and clarity of the table, which is focused on continuous demographic, anthropometric, and functional variables, and their distributions between male and females. Including categorical data such as ethnicity would require reformatting the table in a way that could compromise readability with respect to the current focus. That said, in agreement with R1.5, we have emphasized this demographic characteristic in our limitations. We hope this provides sufficient context while preserving the clarity of Table 1.

4. We thank the reviewer for the positive comment on Figure 2.

5. We thank the reviewer for the suggestion. We have included statements of intent throughout the methodology. Specifically we have added statements of intent to:

   Section 2.1.2: Figure 2 provides an overview of our CMR segmentation and preprocessing pipeline. This process was aimed at obtaining consistent, high-resolution segmentation of the cardiac structures of interest, required for the downstream shape analysis; it can be summarized in three steps.

   Section 2.1.3: Following segmentation preprocessing, we generated a surface mesh of the biventricular myocardium, which provides a connected and spatially coherent representation of the cardiac anatomy.

   Section 2.3: We apply principal component analysis (PCA) to the mapped cardiac meshes to retrieve a compact representation of the anatomical variability in our studied cohort, and a set of low-dimensional shape descriptors that could be subsequently used to quantify and compare morphological patterns across individuals.

   Section 2.4.1: To assess whether the overall cardiac shape differs systematically between males and females, we conducted a sex-specific analysis comparing the distributions of shape

coefficients. We employ Hotelling's T-squared test to determine whether the multivariate distributions of *sex-stratified* shape coefficients differ significantly between the male and female subgroups.

Section 2.4.2: To investigate how cardiac shape variation is influenced by sex and other potential confounders, we employed multivariate analysis of covariance (MANCOVA). MANCOVA extends the analysis of covariance and regression by assessing multiple dependent variables against both categorical and continuous independent variables.

Section 2.4.3: To isolate the effects of sex on cardiac shape from those of other influencing variables, we performed a correction step to remove the impact of significant confounders from the shape coefficients. After identifying significant confounders, we used multivariate linear regression to correct the shape coefficients for these variables, excluding sex.

Section 2.4.4: To evaluate the discriminative power of shape coefficients and assess the impact of confounder correction on sex classification, we performed logistic regression analysis. We trained multiple logistic regression models to determine how well corrected and uncorrected shape coefficients can differentiate between male and female subjects. This analysis estimates the probability that a given subject belongs to a particular class (male or female), based on the values of the independent variables (corrected and uncorrected shape coefficients).

6. We thank the reviewer for the relevant comment. Both equal variance and normality were checked, further info about the relevant tests have been provided in sections 2.4.1 and 2.4.2 of the manuscript:

   We conducted the Hotelling's T-squared test both before and after correcting for confounders to evaluate whether sex-specific differences in the shape coefficients remain significant. In all analyses, equality of variance between the groups was assessed through Levene's test and a p-value below 0.05 is considered statistically significant.

   Normality was assessed through Kolmogorov-Smirnov test and a p-value below 0.05 is considered statistically significant for the results of the analysis.

7. We thank the reviewer for the comment on Figure 4. We believe that with the added explanation on the template optimization (following comment R1.3), Figure 4 now nicely shows the difference between the initial (idealized) template, and final (population-driven) template.

8. We thank the reviewer for the supportive comment on Figure 5.

9. We appreciate the reviewers suggestion with respect to Figure 6. We fully agree that the visualization of the modes is not entirely clear as the figure currently stands. Hence, we have modified the figure to show only the first 4 modes. The first 8 modes are still considered for visualization purposes, but are instead provided as animations in the supplementary material, as previously mentioned in R1.2).

10. We appreciate the reviewers comment on Figure 7 and have addressed it as follows:

    (a) We have expanded the description of Figure 7, please refer to the response to R1.2 above.

    (b) We have added further annotation on the figure, namely star symbols for significance.

    (c) We removed the black bars and mean points.

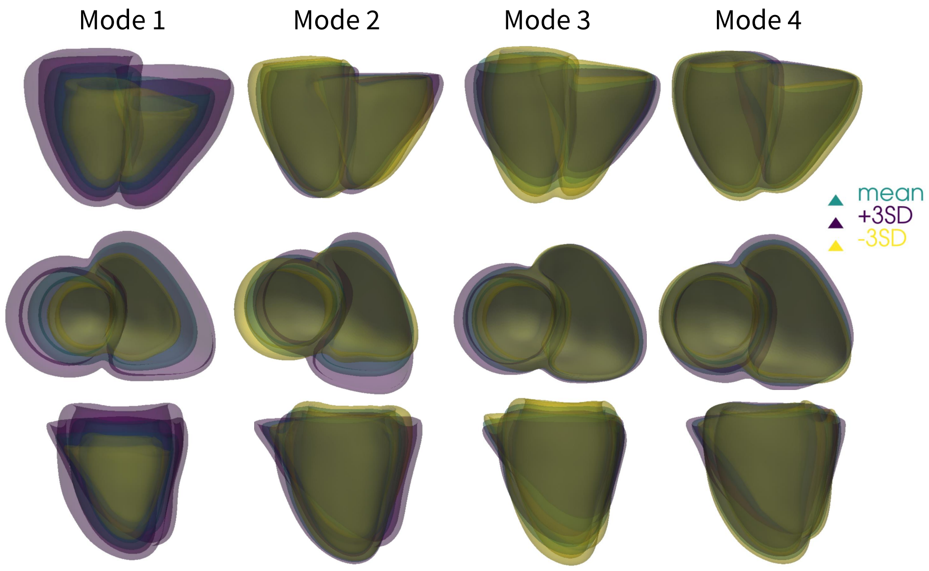

Figure 6: **The first four modes of anatomical variation of the biventricular cardiac anatomy in the cohort**. For each mode, we display overlay of the mean shape and the shape at the +/-3 standard deviation (SD).

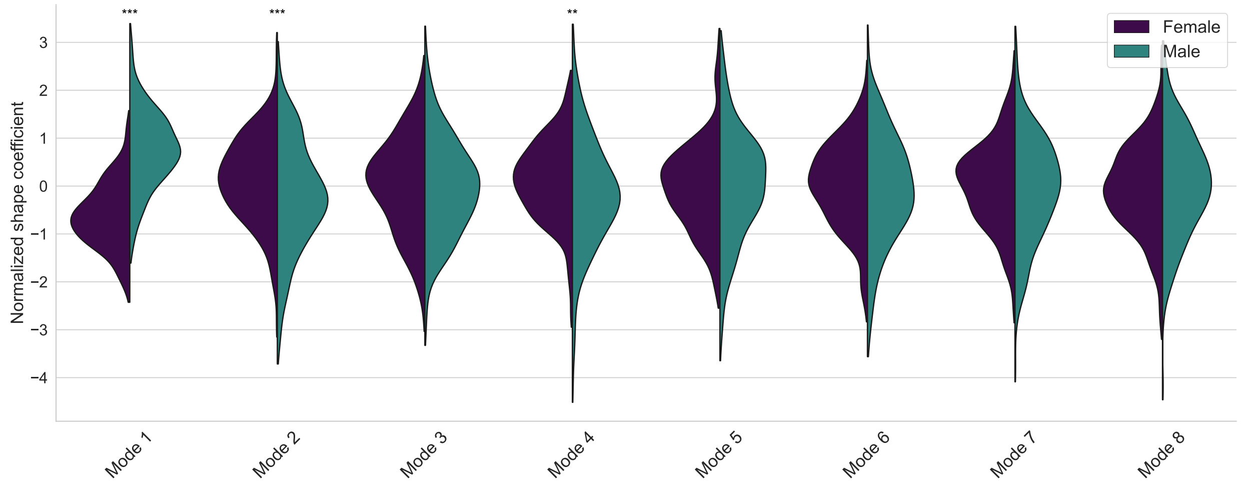

Figure 7: **Distribution of the shape coefficients for the first eight modes, stratified by sex.** Purple represents females and teal represents males. The shape coefficients are provided as z-scores normalized by their mean and standard deviation.Asterisks above the violins indicate statistical significance of the difference between male and female distributions based on an independent t-test: * indicates $p < 0.05$, ** indicates $p < 0.01$, *** indicates $p < 0.001$.

11. We appreciate the reviewer's comment on the Figure 8. We have introduced the following revisions to improve the clarify of the figure:

    - We have modified the caption to explain more clearly the insights provided by Pillai's

trace:

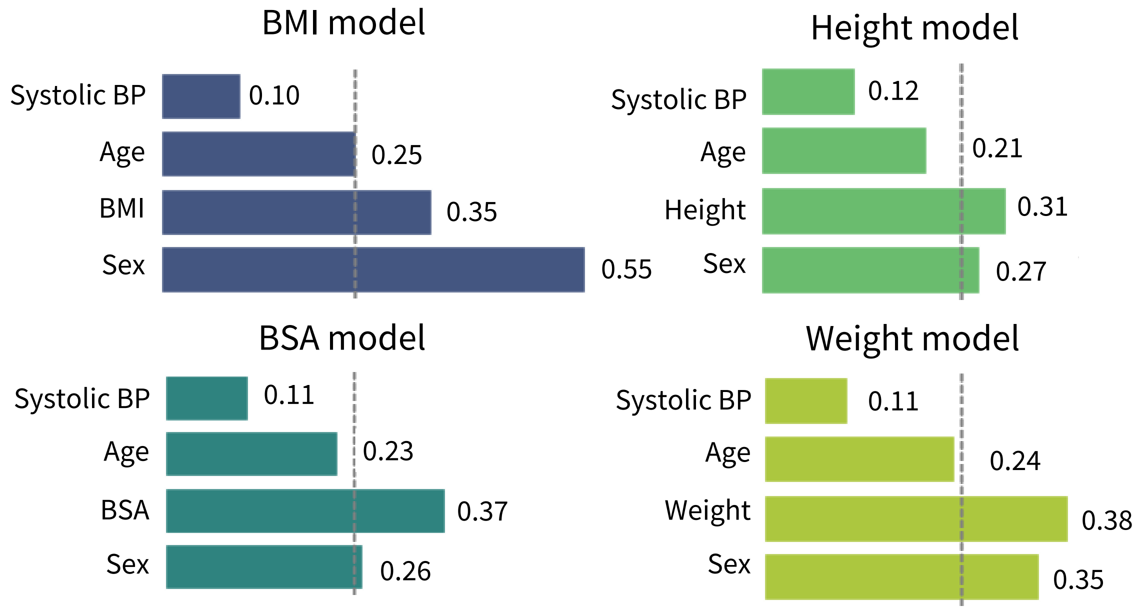

Figure 8: **Contribution of explanatory variables to cardiac shape variability among the four different models.** Each bar represents Pillai's trace, which quantifies the amount of variance in the shape coefficients attributed by each explanatory variable after correction for the other confounders. It is calculated as the sum over all predictors p, of the fraction $\lambda_p/(1+\lambda_p)$, where λ_p are the eigenvalues of the matrix $\mathbf{A} = \mathbf{HE}^{-1}$, with $\mathbf{H}$ and $\mathbf{E}$ being the model sum of squares and residual sum of squares matrices, respectively. A value of 0 indicates no contribution of the explanatory variable to cardiac shape variability, while a value closer to 1 suggests a strong effect. All explanatory variables have a statistically significant influence on morphological variability, and sex explains at least 25% of the variability in all models.

- We have fixed the x-axis to a common range (x in [0, 0.6]) to make comparisons more intuitive between models, and added a dash vertical line at 0.25.
- We have added subtitles to each model to improve clarify on which models the results are pointing to.

  With respect to the sex-specific progression of heart shape with age, we fully agree that it is a very interesting point of investigation. As such , we have included it in our outlook as follows:

  In addition, longitudinal studies could elucidate how sex-based anatomical differences evolve with aging and/or disease progression.

12. We thank the reviewer for the remarks and suggestions on tables 2 & 3. We will hereby clarify the interpretation of Table 2 & 3:

    With respect to Table 2: the results of the Hotelling's t-squared test show that significant sex differences in the multivariate distribution of shape coefficients are present, after correcting for body size and the other confounders. While cardiac size (i.e., mode 1) plays a role in this, these results highlight that this is the case regardless of body size.

With respect to Table 3: the pseudo-R2 indicates the goodness-of-fit of the model, and hence the power of cardiac morphology in discriminating men and women, while the Likelihood Ratio Test p-value indicates the statistical significance of the regression model. Overall, the results in this table indicate that, regardless of the correction scheme, the shape coefficients alone allow significant discrimination between men and women. However, we do agree that this information is qualitatively similar to the ROC and AUC values provided in Figure 9, and have therefore decided to move the table to Appendix E.

13. We thank the reviewer for their comments and suggestions on Figure 10 and Table 4. We addressed each point as follows:

    - Complementary to the power of discrimination of the whole ensemble of shape coefficients, Table 4 reports the logistic regression coefficients of each mode separately, indicating which modes significantly contribute to the discrimination in the models. As such, it shows that while the main driver of discrimination is mode 1 (cardiac size), other anatomical modes also significantly contribute to the discrimination. Figure 10 complements the results with visualization of these discriminative anatomical modes of variation.
    - We agree that visualizing all the five models can make the figure more complex to visualize - however, one of our aims in this visualization was to showcase how the different correction schemes reveal different discriminative patterns, as well as how all models have common discriminative patterns that persist after correction.
    - The gray anatomy in Mode 7, as well as in the other visualization, indicates no statistical differences - regardless of the orientation of the other anatomies in the same column.

Response To Reviewer #2

Overall Comments

> Sex has been increasingly recognized as an independent biological variable influencing cardiovascular disease risk. While the link between sex and cardiac anatomical features has been established, studies quantitatively assessing its impact independent of common confounders-such as age and body metrics-remain scarce. In this study, Drs. Moscoloni and colleagues conducted a computational analysis using statistical shape modeling to investigate sex-based differences in healthy cardiac anatomy. Their results demonstrate that sex explains at least 25% of anatomical variability, primarily affecting cardiac chamber volume, the anterior-posterior width of the right ventricle, and the relative positioning of the chambers. These findings provide valuable insights into the role of sex in cardiac structure.
>
> Overall, the study's methodology is well justified, and the manuscript presents a reasonable amount of data supporting its conclusions. However, some limitations should be noted: 1) Narrow confounder adjustment - The analysis did not account for other potential confounders beyond age and body size. 2) Methodological clarity - Some descriptions of the analytical approach are unclear. 3) Readability - The highly quantitative nature of the study makes the manuscript challenging to follow in places. These weaknesses are explained as follows.

Response

We would like to thank the reviewer for the great summary of our work, and for the valuable feedback and remarks on our manuscript. We have carefully addressed the limitations that have been raised up, and address the comments. We hope that the implemented changes and revised version of the manuscript is satisfactory.

Reviewer Comment R2.1

> The analysis examines associations between biventricular shape statistics and factors such as sex, age, blood pressure, and body metrics. However, the inclusion of covariates appears insufficient. Notably, lifestyle factors (e.g., smoking, alcohol use) - known risk modifiers for cardiac pathology - were not incorporated. Clarifying whether such variables influence shape statistics would strengthen the interpretation of sex-specific effects and improve clinical relevance.

Response

We thank the reviewer for this remark on the choice of the covariates to include. We fully acknowledge that we focused on a restricted amount of covariates in our analysis and did not include covariates that constitute risk modifiers for cardiac pathology. However this was a precise study design, given that our primary aim was to focus on healthy subjects in our current analysis. As

mentioned in Section 2.1.1, we applied multiple selection criteria that would allow to rule out the effect of risk factors on cardiac morphology:

"From these, we selected 456 healthy subjects with no reported history of cardiovascular disease, hypertension, respiratory disease, diabetes mellitus, hyperlipidemia, hematologic disease, renal disease, rheumatologic disease, malignancy, symptoms of chest pain or dyspnea (Petersen et al. 2016). To rule out additional risk factors, we further excluded current or former smokers and individuals with body mass index (BMI) $\geq$ 30 kg/m."

Nonetheless, we acknowledge the importance of looking at the sex-specific effects of risk factors on cardiac morphology, as well as the effect of other and as such we included this important point in our limitations:

Limitations. While this study presents a novel statistical shape analysis to investigate sex differences in cardiac anatomy, several limitations should be acknowledged. First, we selected a reasonably large cohort of healthy subjects from the UK Biobank population, enabling us to focus on baseline anatomical sex differences. However, our analysis was restricted to individuals of Caucasian ethnicity, which may limit the generalizability of our results to other ethnic groups. Extending the analysis to a larger and more diverse population that includes different ethnic backgrounds, would enhance the applicability of our findings. Second, although we corrected for key demographic and physiological confounders (e.g., age, body size, systolic blood pressure), unmeasured variables such as physical activity, environmental factors, or socioeconomic status may still influence the observed shape differences. As such, a dedicated analysis including a broader range of confounding factors would further improve our understanding of sex differences in cardiac morphology.

Reviewer Comment R2.2

> While MANCOVA was appropriately chosen to assess shape coefficient associations, its validity depends on the normality of dependent variables. The manuscript does not explicitly state whether normality testing was performed or how potential violations were addressed. Documentation of these methodological checks would enhance the robustness of the statistical approach.

Response

The authors thank the reviewer for the important remark on assumption testing. We assessed the normality of the shape coefficients using the Kolmogorov-Smirnov test. We have added a statement about the methodological checks in subsection 2.4.2:

We performed four separate MANCOVA tests, each investigating the effect of sex, age, systolic blood pressure, and a different respective body size metric as a predictor: body mass index (*BMI-model*), body surface area (*BSA-model*), height (*height-model*), and weight (*weight-model*). For each model, we evaluated Pillai's trace. Normality was assessed through Kolmogorov-Smirnov test and a p-value below 0.05 is considered statistically significant for the results of the analysis.

Reviewer Comment 2.3

The manuscript in its current form appears hard to understand. This is partially because the study is very quantitative with several different analytical approaches that are not routinely performed in physiology. For example, given that the statistical shape analysis is the core analytical approach in this study, some intermediate steps might be helpful considering the broad readership of this journal. Also, in multiple occasions descriptions of the approach appear unclear. Some examples include:

1. Page 4, Step 2: Motion correction is mentioned but not clearly described. In addition, it is unclear whether the beta-VAE approach implements motion correction in addition to up-sampling. Some descriptions in Methods appears unclear and could benefit from figure illustrations. For instance, while a region of interest was defined to standardize the subsequent analysis, only landmark descriptions are provided for Step 3.
2. Some approaches may warrant more detailed descriptions of their justifications. For example, the minimization of the cost function in Eq. 2 was achieved through a two-step iteration (page 6). However, it remains unclear whether these steps were adapted from previous studies without modifications or improved by the authors, and why these particular approaches were chosen.
3. For the LDDMM approach described on page 6: Are the results of the anatomical mapping and reconstruction and the subsequent analysis dependent on the original input "template"? While Eq. 5 describes an average template, it is unclear how this template was obtained. Was it generated through the two-step optimization described under Eq. 2? More consistent descriptions would help the reviewer better follow the methodology. Additionally, note that Γ_0 represents the 'derived' template in Fig. 4. The manuscript would benefit from more consistent and distinguishable notation for "template" at different processing steps.
4. The statement: "Of the top 10 hyperparameter combinations evaluated, we qualitatively based our choice on the trade-off between low reconstruction error, avoiding too local and unrealistic deformations, and limiting run time, arriving at $\lambda_v = 10$ mm and $\lambda_W = 6$ mm" could be strengthened with examples or illustrations to help readers understand why/how these specific hyperparameters were optimal.

Response

We thank the reviewer for the remarks valuables points where the clearness of our manuscript can be improved. We have addressed the remarks point my point as follows:

1. **Page 4, Step 2 - Motion correction**: We thank the reviewer for pointing this out. We have revised the manuscript to clarify that the beta-VAE framework we use performs both motion correction and resolution enhancement in an integrated manner, within the latent optimization process. Specifically, the model described in (Wang et al. 2021) is trained to learn manifolds of anatomically plausible high-resolution segmentations using a generative model, and then mapping points from the low-dimensional latent space Z to the space of high-resolution segmentations. When given a low-resolution segmentation, the model infers

the high-resolution segmentation by optimizing both latent vector z, which determines the anatomical shape, and a set of motion displacement parameters. These displacements correspond to slice-wise misalignment typically caused by respiratory or cardiac motion (Pentenga, Puck and Stroh, Ashley and van Genuchten, Wouter and Helbing, Wim A. and Peirlinck, Mathias 2023). We clarified the contribution of the beta-VAE model as follows, and refer to the original work for further details on the training of the network:

We process the CMR segmentations to apply motion correction and enhance spatial resolution, making them more suitable for shape analysis. To this end, we use an openly available latent optimization framework (Wang et al. 2021) trained on a research cohort of cardiac super-resolution label maps (spatial resolution $1.25 \times 1.25 \times 2\ \text{mm}^3$) from 1,200 healthy volunteers. This β-variational autoencoder (VAE) approach performs both resolution enhancement and motion correction by inferring a manifold of anatomically-plausible high-resolution segmentations. The model then leverages a multiview latent optimization process to iteratively infer the high-resolution segmentation corresponding to a low-resolution, motion-degraded input segmentation. The process allows up scaling the $1.25 \times 1.25 \times 8\,\text{mm}^3$ spatial resolution of a routine CMR segmentation, to the $1.25 \times 1.25 \times 2\,\text{mm}^3$ resolution of the training label maps (Wang et al. 2021). For further details about the pretrained networks, we refer the interested reader to the original work (Wang et al. 2021).

2. **Page 4, Step 3 - Region of interest**: We thank the reviewer for this helpful suggestion. We have revised both the methods section and the appendix to clarify how we derive and apply patient-specific cutting planes based on atrial landmarks. Specifically we modified Section 2.2.1 as follows:

   Step 3 - region of interest and RV epicardium estimation. We refine the output segmentations from the β-VAE model using atrial landmarks, i.e., the mitral and tricuspid valve planes, derived from the long-axis atrial blood pool segmentations (Bai et al. 2018). Each landmark was used to define a cutting plane that is approximately orthogonal to the short-axis view. The region of interest was defined as the voxels from left and right ventricles that fall below the mitral and tricuspid planes, respectively. The construction of these planes is detailed in Appendix A.

   Additionally, we added a summary of the steps taken to define the region of interest, starting from the identification of the landmarks, in the appendix:

   To define standardized regions of interest for our shape analysis, we derive patient-specific cutting planes based on the anatomical positions of the mitral and tricuspid valves, approximated using long-axis atrial segmentations. The cutting process involves: (i) identifying the atrioventricular junction via directional analysis of the atrial label; (ii) constructing a cutting plane through this landmark; and (iii) retaining only the voxels below this plane in each ventricle.

3. **Page 6, Eq. 2 - Two-step optimization**: We thank the reviewer for pointing out the need for more clarity. The minimization of the cost function via a two-step iterative scheme is a well-established strategy in the LDDMM framework, as implemented in the open-source software Deformetrica (Bône et al. 2018), and originally proposed by Durrleman et al. for morphometric analysis using dense diffeomorphic deformations (Durrleman et al. 2014). In our study, we adopted this optimization strategy without modifications, and we now clarify the practical goal of each optimization step in our revised text as follows:

To optimize Eq. 1, we iterate until convergence (Bône et al. 2018): (i) Optimization with respect to the momenta $\boldsymbol{\mu}$ while keeping the template $\mathbf{\Gamma}$ fixed, yielding the $N_{\mathcal{P}}$ deformations that map the (current) template to each target geometry. (ii) Optimization with respect to the template $\mathbf{\Gamma}$ while keeping the momenta $\boldsymbol{\mu}$ fixed, resulting in an updated template derived from the (current) set of subject-specific momenta. The first step computes the subject-specific deformations needed to match the template to each target anatomy, establishing point-to-point correspondence between the template and the target mesh. The second step ensures that the final template reflects population-wide anatomical baseline, facilitating both physiological interpretability and more accurate shape modeling. For further details about the LDDMM framework and the optimization approach we refer to the original work (Durrleman et al. 2014; Bône et al. 2018). Once optimized, the cohort of geometries is represented by the final template $\mathbf{\Gamma}$ and a unique set of momenta $\boldsymbol{\mu}_i$ for each subject, i.e., target geometry i. By applying the momenta to the template vertices, the corresponding biventricular surface meshes are reconstructed for each subject, ensuring consistent point-to-point correspondence across all meshes in the cohort.

In a broader sense, we selected LDDMM for this application due to its ambient-space formulation, which ensures that deformations are smooth and topology-preserving. This makes it particularly suitable for anatomical shape mapping tasks where the integrity of the anatomy must be maintained throughout the mapping process.

4. **Page 6 - LDDMM Template**: We thank the reviewer for this important observation. The results of the anatomical mapping and subsequent shape analysis are indeed influenced by the choice of the initial input template, especially at the start of the optimization process. However, due to the iterative nature of the process, the influence of the initial geometry (i.e., in our case, an idealized biventricular geometry) diminishes as the template is updated across iterations to reflect the cohort-wide anatomical average, until convergence to the population average. We agree that the previous description could have been more consistent, and in line with the response to the previous point, we have clarified the need to iteratively update the template in Section 2.2.1.

   With to the caption of figure 4, the term "derived" template was referring to the process of deriving it from the complete heart scaffold (Section 2.2.2); yet, we fully agree this creates confusing with the rest of the description of the optimization process and have hence renamed it to "initial template" in the caption of Figure 4:

   Anatomical mapping of the biventricular surface meshes. (a) The initial biventricular template $\mathbf{\Gamma_0}$ embedded in the ambient space. (b) Final population template $\mathbf{\Gamma}$ after mapping.

5. **Page 7 - Hyperparameter optimization**: We thank the reviewer for the helpful suggestion. We agree that a clearer explanation of the hyperparameter choice would aid in understanding. To avoid overloading the manuscript with additional figures, we have opted to strengthen the text with more concrete justification and explanation on the choice of the parameters:

   Of the top 10 hyperparameters combinations evaluated, we qualitatively base our choice on the following criteria: (i) reconstruction accuracy, measured via the total reconstruction error, (ii) anatomical plausibility, assessed by visual inspection to detect overfitting or local unrealistic warping, and (iii) acceptable computational runtime. The trade-off between these criteria led to $\lambda_V = 10$ mm and $\lambda_W = 6$ mm.

Reviewer Comment R2.4

> The authors are encouraged to have a data/code availability statement/sharing plan should this manuscript be revised.

Response

We thank the reviewer for the very important remark. We have added a data/code availability statement in the Additional information, as follows:

The raw imaging data and non-imaging participant characteristics are available from UK Biobank to approved researchers via a standard application process at http://www.ukbiobank.ac.uk/register-apply. Scripts for the shape modeling and analysis will be made available upon publication at https://github.com/peirlincklab/cardiacshapemodeling.

Reviewer Comment R2.5

> Minor comments:
>
> - Eq. 3, 1/N should be $1/N_v$ instead?
> - Eqs. 5 and 6 both describeα_i, however they seem to represent distinct variables. Thus it would be helpful to distinguish the two by using different subscripts.
> - In Fig 4, how different quantitatively are those two templates?
> - Fig 8, the 'four' different models should be labelled explicitly.
> - Please describe how pseudo-R2 was computed in the logistic regression analysis, given that multiple ways of pseudo-R2 have been documented.

Response

We thank the reviewer for the useful comments. Each point is addressed as follows:

- We have corrected the denominator of Eq. 3 from N to N_v, as, in fact, it is referring to the number of vertices. The corrected equation now reads:

$$\ell_i = \frac{1}{N_v} \sum_{j=1}^{N_v} \|\mathbf{p}(j)_i - \mathbf{r}(j)_i\| \tag{2}$$

- α_i in the two equations does indeed represent different variables. We have hence renamed the portion of shape coefficient attributed to other sources as α_I, and Eq. 6 now reads as follows:

$$\alpha = \alpha_M + \alpha_I + \epsilon \tag{3}$$

- As explained in text, the two templates shown in Fig. 4 are substantially different, as the initial (generalized template) gets iteratively updated to converge to the "real" population-average template.

- We have updated Fig. 8 with explicit labels.

- We clarified that pseudo-R^2 values reported in the logistic regression analysis correspond to McFadden's pseudo-R^2, as implemented by the *statsmodels* package. This measure compares the log-likelihoods of the full model and a null model, providing an indication of model fit. We have also added a reference to McFadden and included a brief explanation in Appendix E (McFadden 1974):

  The pseudo-R^2 is calculated within the *statsmodel* package according to McFadden's pseudo-R-squared formulation, where the pseudo-R^2 is based on the ratio between the log-likelihood of the fitted model and that of a null model (a model with only an intercept). Specifically, it reflects the proportional improvement in model fit over the null model, with higher values indicating better explanatory power (McFadden 1974).

References

Bai, W., M. Sinclair, G. Tarroni, O. Oktay, M. Rajchl, G. Vaillant, A. M. Lee, N. Aung, E. Lukaschuk, M. M. Sanghvi, F. Zemrak, K. Fung, J. M. Paiva, V. Carapella, Y. J. Kim, H. Suzuki, B. Kainz, P. M. Matthews, S. E. Petersen, and S. K. Piechnik (Sept. 2018). "Automated cardiovascular magnetic resonance image analysis with fully convolutional networks". In: *Journal of Cardiovascular Magnetic Resonance* 20.1.

Bône, A., M. Louis, B. Martin, and S. Durrleman (Sept. 2018). "Deformetrica 4: an open-source software for statistical shape analysis". In: 3–13.

Bruse, J. L., K. McLeod, G. Biglino, H. N. Ntsinjana, C. Capelli, T.-Y. Hsia, M. Sermesant, X. Pennec, A. M. Taylor, S. Schievano, A. Taylor, A. Giardini, S. Khambadkone, S. Schievano, M. d. Leval, T. Y. Hsia, E. Bove, A. Dorfman, G. H. Baker, A. Hlavacek, F. Migliavacca, G. Pennati, G. Dubini, A. Marsden, J. Feinstein, I. Vignon-Clementel, R. Figliola, and J. McGregor (2016). "A statistical shape modelling framework to extract 3D shape biomarkers from medical imaging data: assessing arch morphology of repaired coarctation of the aorta". In: *BMC Medical Imaging* 16.1, p. 40.

Burns, R., W. J. Young, N. Aung, L. R. Lopes, P. M. Elliott, P. Syrris, R. Barriales-Villa, C. Sohrabi, S. E. Petersen, J. Ramírez, A. Young, and P. B. Munroe (2024). "Genetic basis of right and left ventricular heart shape". In: *Nature Communications* 15.1, p. 9437.

Durrleman, S., M. Prastawa, N. Charon, J. R. Korenberg, S. Joshi, G. Gerig, and A. Trouvé (Nov. 2014). "Morphometry of anatomical shape complexes with dense deformations and sparse parameters". In: *NeuroImage* 101, pp. 35–49.

Gao, Z., Z. Chen, A. Sun, and X. Deng (2019). "Gender differences in cardiovascular disease". In: *Medicine in Novel Technology and Devices* 4, p. 100025.

Govil, S., C. Mauger, S. Hegde, C. J. Occleshaw, X. Yu, J. C. Perry, A. A. Young, J. H. Omens, and A. D. McCulloch (2023). "Biventricular shape modes discriminate pulmonary valve replacement in tetralogy of Fallot better than imaging indices". In: *Scientific Reports* 13.1, p. 2335.

Kruk, E. van der (Mar. 2025). "BIASMECHANICS: Does an unconscious bias still persist in biomechanics, positioning males as the default in human research? A meta-analysis on the Journal of Biomechanics 2024 publications". In: *Journal of Biomechanics* 181, p. 112560.

Lin, H., A. C. Kwan, C. Castro-Diehl, M. I. Short, V. Xanthakis, I. M. Yola, G. Salto, G. F. Mitchell, M. G. Larson, R. S. Vasan, and S. Cheng (2023). "Sex-specific differences in the genetic and environmental effects on cardiac phenotypic variation assessed by echocardiography". In: *Scientific Reports* 13.1, p. 5786.

Littlejohns, T. J., J. Holliday, L. M. Gibson, S. Garratt, N. Oesingmann, F. Alfaro-Almagro, J. D. Bell, C. Boultwood, R. Collins, M. C. Conroy, N. Crabtree, N. Doherty, A. F. Frangi, N. C. Harvey, P. Leeson, K. L. Miller, S. Neubauer, S. E. Petersen, J. Sellors, and S. Sheard (May 2020). "The UK Biobank imaging enhancement of 100,000 participants: rationale, data collection, management and future directions". In: *Nature Communications* 11.1.

Martin, T. G. and L. A. Leinwand (2024). "Hearts apart: sex differences in cardiac remodeling in health and disease". In: *Journal of Clinical Investigation* 134.13, e180074.

Martinez, E., B. Moscoloni, M. Salvador, F. Kong, M. Peirlinck, and A. L. Marsden (2025). *Full-field surrogate modeling of cardiac function encoding geometric variability.*

McFadden, D. (1974). *Conditional logit analysis of qualitative choice behavior.* Tech. rep. 105-142. New York: Frontiers in Econometric.

Mincholé, A., J. Camps, A. Lyon, and B. Rodríguez (2019). "Machine learning in the electrocardiogram". In: *Journal of Electrocardiology* 57, S61–S64.

Peirlinck, M., F. S. Costabal, J. Yao, J. M. Guccione, S. Tripathy, Y. Wang, D. Ozturk, P. Segars, T. M. Morrison, S. Levine, and E. Kuhl (Feb. 2021). "Precision medicine in human heart modeling: Perspectives, challenges, and opportunities". In: *Biomechanics and Modeling in Mechanobiology* 20.3, pp. 803–831.

Peirlinck, M., J. Yao, F. Sahli Costabal, and E. Kuhl (Mar. 2022). "How drugs modulate the performance of the human heart". In: *Computational Mechanics* 69.6, pp. 1397–1411.

Peirlinck, M., F. S. Costabal, and E. Kuhl (2021). "Sex differences in drug-induced arrhythmogenesis". In: *Frontiers in Physiology* 12, p. 708435.

Pentenga, Puck and Stroh, Ashley and van Genuchten, Wouter and Helbing, Wim A. and Peirlinck, Mathias (2023). "Shape Morphing and Slice Shift Correction in Congenital Heart Defect Model Generation". In: *Functional Imaging and Modeling of the Heart.* Ed. by O. Bernard, P. Clarysse, N. Duchateau, J. Ohayon, and M. Viallon. Cham: Springer Nature Switzerland, pp. 347–355.

Petersen, S. E., N. Aung, M. M. Sanghvi, F. Zemrak, K. Fung, J. M. Paiva, J. M. Francis, M. Y. Khanji, E. Lukaschuk, A. M. Lee, V. Carapella, Y. J. Kim, P. Leeson, S. K. Piechnik, and S. Neubauer (2016). "Reference ranges for cardiac structure and function using cardiovascular magnetic resonance in Caucasians from the UK Biobank population cohort". In: *Journal of Cardiovascular Magnetic Resonance* 19.1, p. 18.

Reue, K. and C. B. Wiese (2022). "Illuminating the mechanisms underlying sex differences in cardiovascular disease". In: *Circulation Research* 130.12, pp. 1747–1762.

Salvador, M., F. Kong, M. Peirlinck, D. W. Parker, H. Chubb, A. M. Dubin, and A. L. Marsden (June 2024). "Digital twinning of cardiac electrophysiology for congenital heart disease". In: *Journal of The Royal Society Interface* 21.215.

Shiwani, H., R. H. Davies, C.-C. Topriceanu, R. Ditaranto, A. Owens, B. Raman, J. Augusto, R. K. Hughes, C. Torlasco, B. Dowsing, J. Artico, G. Joy, I. Miranda, W. Witschey, J. F. Rodriguez-Palomares, C. Badia-Molins, L. Crotti, M. Cortina-Borja, M. L. Chuang, R. Y. Kwong, C. M.

Kramer, W. Manning, C. Y. Ho, P. Kellman, A. D. Hughes, E. Biagini, S. Mohiddin, L. Lopes, H. Litt, V. A. Ferrari, G. Captur, J. C. Moon, H. Shiwani, R. H. Davies, C.-C. Topriceanu, R. Ditaranto, A. Owens, B. Raman, J. Augusto, R. K. Hughes, C. Torlasco, B. Dowsing, J. Artico, G. Joy, I. Miranda, W. Witschey, J. F. Rodriguez-Palomares, C. Badia, L. Crotti, M. Cortina-Borja, M. L. Chuang, R. Y. Kwong, C. M. Kramer, W. Manning, C. Y. Ho, P. Kellman, A. D. Hughes, E. Biagini, S. Mohiddin, L. Lopes, H. Litt, V. Ferrari, G. Captur, J. C. Moon, R. Bachiller, V. Zaha, R. M. Peshock, H. H. Hobbs, A. Shah, S. Rosmini, S. Castelletti, I. Pierce, K. Moschonas, M. Webber, S. Sivalokanathan, J. Malcolmson, E. Thompson, L. Lovato, and A. Ponziani (2025). "Demographic-Based Personalized Left Ventricular Hypertrophy Thresholds for Hypertrophic Cardiomyopathy Diagnosis". In: *Journal of the American College of Cardiology* 85.7. Special Focus Issue: Heart Failure, pp. 685–695.

St. Pierre, S. R., B. Kaczmarski, M. Peirlinck, and E. Kuhl (Apr. 2024). "Sex-specific cardiovascular risk factors in the UK Biobank". In: *Frontiers in Physiology* 15.

Suinesiaputra, A., P. Ablin, X. Alb, M. Alessandrini, J. Allen, W. Bai, S. imen, P. Claes, B. R. Cowan, J. Dhooge, N. Duchateau, J. Ehrhardt, A. F. Frangi, A. Gooya, V. Grau, K. Lekadir, A. Lu, A. Mukhopadhyay, I. Oksuz, N. Parajuli, X. Pennec, M. Pereaez, C. Pinto, P. Piras, M.-M. Roh, D. Rueckert, D. Sring, M. Sermesant, K. Siddiqi, M. Tabassian, L. Teresi, S. A. Tsaftaris, M. Wilms, A. A. Young, X. Zhang, and P. Medrano-Gracia (2018). "Statistical shape modeling of the left ventricle: myocardial infarct classification challenge". In: *IEEE Journal of Biomedical and Health Informatics* 22.2, pp. 503–515.

Wang, S., C. Qin, N. Savioli, C. Chen, D. P. O'Regan, S. A. Cook, Y. Guo, D. Rueckert, and W. Bai (Sept. 2021). "Joint motion correction and super resolution for cardiac segmentation via latent optimisation". In: *Lecture Notes in Computer Science*, pp. 14–24.

Westaby, J. D., E. Zullo, L. M. Bicalho, R. H. Anderson, and M. N. Sheppard (2023). "Effect of sex, age and body measurements on heart weight, atrial, ventricular, valvular and sub-epicardial fat measurements of the normal heart". In: *Cardiovascular Pathology* 63, p. 107508.

Winham, S. J., M. d. Andrade, and V. M. Miller (2015). "Genetics of cardiovascular disease: Importance of sex and ethnicity". In: *Atherosclerosis* 241.1, pp. 219–228.

Dear Professor Peirlinck,

Re: JP-RP-2025-288667R1 "Unveiling sex dimorphism in the healthy cardiac anatomy: fundamental differences between male and female heart shapes" by Beatrice Moscoloni, Cameron Beeche, Julio A. Chirinos, Patrick Segers, and Mathias Peirlinck

We are pleased to tell you that your paper has been accepted for publication in The Journal of Physiology.

The last Word (or similar) version of the manuscript provided will be used by the Production Editor to prepare your proof. When this is ready you will receive an email containing a link to Wiley's Online Proofing System. The proof should be thoroughly checked and corrected as promptly as possible.

Authors should note that it is too late at this point to offer corrections prior to proofreading. Major corrections at proof stage, such as changes to figures, will be referred to the Editors for approval before they can be incorporated. Only minor changes, such as to style and consistency, should be made at proof stage. Changes that need to be made after proof stage will usually require a formal correction notice.

All queries at proof stage should be sent to: TJP@wiley.com.

Following copyediting and typesetting the accepted version will be published online.

Yours sincerely,

Bjorn Knollmann
Senior Editor
The Journal of Physiology

IMPORTANT POINTS TO NOTE FOLLOWING YOUR PAPER'S ACCEPTANCE:

If you would like to receive our 'Research Roundup', a monthly newsletter highlighting the cutting-edge research published in The Physiological Society's family of journals (The Journal of Physiology, Experimental Physiology, Physiological Reports, The Journal of Nutritional Physiology and The Journal of Precision Medicine: Health and Disease), please click this link, fill in your name and email address and select 'Research Roundup':
https://www.physoc.org/journals-and-media/membernews

• TRANSPARENT PEER REVIEW POLICY: To improve the transparency of its peer review process, The Journal of Physiology publishes online as supporting information the peer review history of all articles accepted for publication. Readers will have access to decision letters, including Editors' comments and referee reports, for each version of the manuscript as well as any author responses to peer review comments. Referees can decide whether or not they wish to be named on the peer review history document.

• You can help your research get the attention it deserves! Check out Wiley's free Promotion Guide for best-practice recommendations for promoting your work at: www.wileyauthors.com/eeo/guide. You can learn more about Wiley Editing Services which offers professional video, design, and writing services to create shareable video abstracts, infographics, conference posters, lay summaries, and research news stories for your research at: www.wileyauthors.com/eeo/promotion.

• IMPORTANT NOTICE ABOUT OPEN ACCESS: To assist authors whose funding agencies mandate public access to published research findings sooner than 12 months after publication, The Journal of Physiology allows authors to pay an Open Access (OA) fee to have their papers made freely available immediately on publication.

The Corresponding Author will receive an email from Wiley with details on how to register or log-in to Wiley Authors Services where you will be able to place an order.

• You can check if your funder or institution has a Wiley Open Access Account here: https://authorservices.wiley.com/author-resources/Journal-Authors/licensing-and-open-access/open-access/author-compliance-tool.html.

EDITOR COMMENTS

Reviewing Editor:

Thank you for addressing all the reviewers' comments. The manuscript is well written and, in my view, ready for publication.

Senior Editor:

The revised manuscript is now acceptable for publication. Thank you for your excellent contribution to the Journal!

REFEREE COMMENTS

Referee #1:

I thank the authors for re-submission of this manuscript. I have reviewed the revised manuscript, and will respond accordingly to the responses the authors have made to each of my previous comments, and their efforts to address them. The revised study improves upon its prior version through the inclusion of additional clarity on methodology and further interpretation providing needed clinical relevance. The insights this manuscript provides are interesting, and contribute towards the fast-growing area of sex-specific differences across cardiovascular systems. This study will inform novel works taking sex-specific morphology into account, and provides useful information which will drive sex-specific analysis of remodeling in upcoming work.

R1.1

The authors have taken the comment regarding the need for additional information on how cardiac biomarkers are affected by variability in shape and size between patients, and they have extended their introduction to include suggested references to recent HCM guidelines (Shiwani et al. 2025) and Minchole et al. 2019. They additionally cite the works of Bruse et al. 2016, on statistical shape modelling of cardiac imaging data and the review by Gao et al. 2019, on gender differences in cardiovascular disease. I am satisfied with their changes to the introduction, and I believe these additions provide a stronger background which further explains the purpose of this work.

Furthermore, in response to my comment that one of the original aims, "to assess the impact of age, body size, BP on morphology" was not adequately evidenced in the prior version of the manuscript, the authors have rephrased this aim to quantify the impact of sex on cardiac morphology after accounting for age, body size and blood pressure. I agree that this aim is much more in line with the focus on sex differences in cardiac morphology, and how co-founding factors (age, BMI, etc.) can be accounted for. I believe this is a significant improvement and I am satisfied with these updates.

R1.2

In the previous version, I believed that the manuscript fell short of interpreting the results of the machine learning and statistical approaches were unsatisfactory. I pointed towards Figures 7 & 8, and Tables 2 & 3 as examples of this. I suggested that some additional descriptions on how the male and female meshes were significantly different from one another in physiological terms would elevate the work and provide physiological and clinical significance.

In response to this, the authors have provided a detailed clarification on the reasoning underlying the current interpretation of the results, and their decision to stay conservative when making clinical interpretations. They explain that these modes may primarily indicate one specific measurement, but carry additional contributions from other measurements, meaning a simplistic "mode 1 = x measurement" would be an oversimplification of the morphological complexity of each mode. Furthermore, a quantitative correlation would require measurements on the individual subject meshes. The authors have expanded the results subsections to expand the physiological differences that each mode suggests, being careful to not oversimplify the results. Additionally, they clarify the implication of Pillai's trace values from MANCOVA analysis with respect to anatomical variance explained by sex, and the practical implication of the Hotelling's T2 test, with respect to sex differences in morphology. This is a responsible and transparent way to address these concerns and I am satisfied with these changes and their reasoning.

R1.3

In response to request for additional clarification on the purpose and relevance of the second step of the cost-minimization algorithm presented in Section 2.2.1, the authors have provided a clarification of the optimization with respect to the initial

template geometry and the need for such a process. This explanation is well reasoned and clear. They have revised section 2.2.1 with these explanations which improves the clarity and fidelity of the manuscript.

R1.4

In response to criticism regarding the comparison with Burns et al. (2024), the authors have acknowledged this and have expanded the discussion on the present study in relation to Burns et al. (2024), and it's much larger sample size. The authors also have added reference to Burns et al. (2024) in the introduction, which I believe further motivates the need for this study, and it's focus on sex-differences accounting for confounding factors (age, body size, shape, etc.). I'm satisfied with these revisions.

R1.5

In response to a request for an expanded limitations section, the authors agreed that limitations needs to be expanded and have revised it to include relevant limitations to both the UKBB cohort, and the selected subcohort used in the study. The new section discusses reasoning for the selection of the subcohort, and acknowledges it's restriction to those of Caucasian ethnicity and potential issues with generalization to other ethnicities. They acknowledge the potential impact of demographic co-founders not included in this study, including socioeconomic status which may additionally impact the results.

They have rephrased the future directions of study, and made it a dedicated section on future outlooks. This expands upon the prior version by discussing the need to bridge studies of this kind with clinically actionable outcomes, and shedding light on how sex-specific morphological differences may inform clinical risk and potentially guide therapeutic response. Similarly, they reference Peirlinck et al. (2021) and St. Pierre et al. (2024), and discuss how applying this framework to disease-specific cohorts would elucidate sex-specific remodeling patterns associated with heart disease.

I believe the authors have addressed this comment, and I would like to make note that the future outlooks section is particularly exciting.

R1.6

In response to a request for further dissection of the results in the discussion, the authors have expanded several sections with more clinically relevant insights. Notably, their suggestion that sex-differences in cardiac anatomy are not limited to average shape, but rather span all morphological aspects is an interesting insight - and highlights the need for sex-specific reference models in both clinical and research settings. Furthermore, additional reference to current diagnostic thresholds, and their need to account for intrinsic sex-differences brings more clinically relevant insights to this work.

In addition to the above, I provided specific comments pertaining to small changes needed in Figures, Tables and text throughout the manuscript. These have all been addressed point by point. Notably, the suggestion for additional statements of the intended outcome of methodological steps was well-addressed, along with adjustments to Figure 7 and 8 which provide needed clarity and context. I would also like to note that the authors have included a data/code availability statement which is much welcomed.

Referee #2:

The reviewer would like to thank the authors for careful consideration of previous comments and suggestions and has no further comments.
